# Task sharing with non-physician health-care workers for management of blood pressure in low-income and middle-income countries: a systematic review and meta-analysis

**DOI:** 10.1016/S2214-109X(19)30077-4

**Published:** 2019-05-13

**Authors:** T N Anand, Linju Maria Joseph, A V Geetha, Dorairaj Prabhakaran, Panniyammakal Jeemon

**Affiliations:** a Centre for Chronic Disease Control, New Delhi, India; b Public Health Foundation of India, New Delhi, India; c London School of Hygiene & Tropical Medicine, London, UK; d Sree Chitra Tirunal Institute for Medical Sciences and Technology, Trivandrum, Kerala, India

## Abstract

**Background:**

Task sharing for the management of hypertension could be useful for understaffed and resource-poor health systems. We assessed the effectiveness of task-sharing interventions in improving blood pressure control among adults in low-income and middle-income countries.

**Methods:**

We searched the Cochrane Library, PubMed, Embase, and CINAHL for studies published up to December 2018. We included intervention studies involving a task-sharing strategy for management of blood pressure and other cardiovascular risk factors. We extracted data on population, interventions, blood pressure, and task sharing groups. We did a meta-analysis of randomised controlled trials.

**Findings:**

We found 3012 references, of which 54 met the inclusion criteria initially. Another nine studies were included following an updated search. There were 43 trials and 20 before-and-after studies. We included 31 studies in our meta-analysis. Systolic blood pressure was decreased through task sharing in different groups of health-care workers: the mean difference was −5·34 mm Hg (95% CI −9·00 to −1·67, *I*^2^=84%) for task sharing with nurses, −8·12 mm Hg (–10·23 to −6·01, *I*^2^=57%) for pharmacists, −4·67 mm Hg (–7·09 to −2·24, *I*^2^=0%) for dietitians, −3·67 mm Hg (–4·58 to −2·77, *I*^2^=24%) for community health workers, and −4·85 mm Hg (–6·12 to −3·57, *I*^2^=76%) overall. We found a similar reduction in diastolic blood pressure (overall mean difference −2·92 mm Hg, −3·75 to −2·09, *I*^2^=80%). The overall quality of evidence based on GRADE criteria was moderate for systolic blood pressure, but low for diastolic blood pressure.

**Interpretation:**

Task-sharing interventions are effective in reducing blood pressure. Long-term studies are needed to understand their potential impact on cardiovascular outcomes and mortality.

**Funding:**

Wellcome Trust/DBT India Alliance.

## Introduction

Low-income and middle-income countries (LMICs) bear a disproportionately large burden of cardiovascular disease, and have fewer resources to address it.^1^ Hypertension, an important risk factor for cardiovascular disease, contributes to more than 10% of disability-adjusted life-years lost in LMICs.^2^ Large randomised controlled trials and prospective observational studies show the benefits of achieving optimal blood pressure control for reducing mortality and cardiovascular outcomes.^3^ However, despite the availability of effective therapies, blood pressure control rates are poor in many LMICs.^4^

LMICs are undergoing an epidemiological transition from predominantly infectious diseases, maternal and child-health conditions, and nutritional disorders to chronic non-communicable diseases such as diabetes and hypertension.^5^ With a rising burden of non-communicable disease, health-policy makers have deliberated the merits of delegating or moving certain tasks from physicians to other health-care professionals, through task shifting or task sharing.^6^ Task shifting is defined as the rational movement of primary care duties from physicians to non-physician health-care workers, such as nurses, pharmacists, or community health workers.^7^ Task sharing is a planned strategy in which a team of health-care professionals work together to deliver a service, accompanied by training or certification and support for health-care workers.^8^, ^9^ Task sharing is considered a more appropriate term than task shifting in highly skilled areas because it is difficult to shift tasks entirely to new cadres of health-care workers.^10^ However, the two terms indicate slightly different scenarios. When there is no physician available, the tasks must be shifted to non-physician health-care workers for the health system to function. When a few physicians are available, tasks may be shared with other health-care professionals with some supervision or referral to physicians.^10^ In LMICs, where access to and availability of physicians can be difficult, utilising the available non-physician health-care workforce may be a logical step for the management of cardiovascular risk. Task sharing in LMICs has been useful in managing maternal and child health^11^ and communicable diseases such as HIV/AIDS.^12^

**Research in context**

**Evidence before this study**

We searched PubMed, Cochrane Library, Embase, and CINAHL without any language restrictions for studies published up to Aug 31, 2017, that described task-sharing interventions for managing blood pressure. We later updated our search to December 2018. Our search terms were related to task sharing and cardiovascular diseases combined with a list of low-income and lower-middle income countries as defined by the World Bank. We identified two reviews of task-sharing interventions for non-communicable diseases. One described the effectiveness of non-physician health-care workers involved in prescription of medications for cardiovascular risk reduction, while the other described enablers and barriers for task-sharing interventions. The reviews show that task sharing is a potentially viable and low-cost strategy for understaffed low-income and lower-middle income countries. We found no published meta-analyses of task-sharing interventions for managing blood pressure.

**Added value of this study**

Our meta-analysis shows that task-sharing interventions are effective in reducing average blood pressure in low-income and lower-middle income countries. Our results validate the possibility of using task sharing for non-communicable disease prevention and management in these settings. However, the impact of task-sharing interventions is greater in countries with better doctor:population ratios. Additionally, we show that interventions are more effective if targeted to high-risk individuals than to the general population.

**Implications of all the available evidence**

Involving non-physician health-care workers in blood pressure control is an effective option in low-income and lower-middle income countries.

Primary and secondary prevention of hypertension, often involve lifestyle counselling, adoption of self-management skills, and the implementation of protocol-led treatment can be instituted by non-physician health-care workers.^13^ A Cochrane review^14^ demonstrated that care led by a nurse or pharmacist could be a favourable way of improving blood pressure control in patients with hypertension, but most of the evidence comes from high-income countries. Such interventions, specifically conducted in LMICs, require further evaluation as effectiveness depends on health system capacity and adaptability. We did a systematic review and meta-analysis of task-sharing interventions and their effects on managing blood pressure in LMICs.

## Methods

### Search strategy and selection criteria

We developed a search strategy based on a previous review^15^ and modified the terms according to the database. We searched PubMed, Embase, Cochrane Library, and CINAHL with terms related to cardiovascular disease, task sharing, and LMICs (appendix pp 46–53). The search covered the period from inception of each database to Aug 15, 2017. We did an updated search up to Dec 28, 2018. We also manually searched the reference lists of identified studies.

We included experimental studies (randomised controlled trials, cluster randomised trials, quasiexperimental studies, and before-and-after designs) that included interventions delivered by community health workers, nurses, pharmacists, and allied health professionals such as dietitians, designed to improve blood pressure control regardless of hypertension status. For the purpose of our review, the tasks shared included non-pharmacological measures such as patient education for lifestyle modification and pharmacological measures such as initiation or refill of prescription medications and titrating the dose of medications. Other measures, such as follow-up and patient reminders for referrals and appointments were also included. The population of interest was adults aged 18 or older, living in LMICs, regardless of their hypertension status.

We did not include studies in which non-physician health-care workers only screened for hypertension. We also excluded studies with patient's knowledge, attitudes, or intentions as outcome variables without measuring any relevant blood pressure outcomes. Peer-led interventions were excluded because they would be more likely to involve informal support. Additionally, we excluded studies of task-sharing activities that are exclusive to traditional healers, alternative therapies such as acupuncture, homoeopathic medicine, and those of only the promotion of self-care or informal caregiver health education.

Furthermore, we excluded studies without before and after measurements of blood pressure, and studies that included fewer than 30 participants or less than 3 months of follow-up from the meta-analyses. Studies with no or insufficient description of randomisation were also excluded from the meta-analyses. We also excluded studies that were not in English.

Our study was registered in the PROSPERO database (CRD42018081015). Ethical approval was not required.

### Data analysis

Initially, duplicates were removed and the remaining citations were reviewed by one author (JLM) based first on the titles to obtain relevant records for abstract screening. All selected abstracts were screened manually and identified relevant articles for full text review by JLM and ATN. Two authors (LMJ and TNA) independently reviewed full texts of all selected studies for final inclusion in the review. Any disagreements were resolved by discussion with a third reviewer (PJ).

Data were extracted when available from published articles. Study authors were contacted twice for data if the outcome of interest was not made available in the published studies. Study quality was assessed in terms of potential bias from randomisation, blinding, outcome assessment, and method of analysis using the Cochrane Risk of bias tool^16^ and National Heart Lung and Blood Institute scale^17^ for before-and-after studies.

Two researchers (LMJ and TNA) cross-checked study details, summary measures, and major outcomes against the published articles. The arbitrator (PJ) reviewed any apparent inconsistencies and made the final recommendation. Blood pressure measurements (in mm Hg) that were done before and after the intervention, for both intervention and control groups, were extracted. We also extracted data for study design, unit of analysis, sample size, study population, task-sharing group, year, author, follow-up duration, country of origin, and intervention details. The main outcomes of interest were changes in systolic and diastolic blood pressure compared with baseline.

### Statistical analysis

We conducted meta-analysis of randomised controlled trials that had at least 30 participants in each group. Although cluster randomised trials were included in the analysis, we estimated effective sample sizes for each study based on their respective design effect.^18^ Design effect was calculated from the reported intraclass correlation coefficient and average cluster size of corresponding studies. Details of the design effect calculation are given in the appendix (pp 53–54).

Net blood pressure was calculated on the basis of the difference between the mean blood pressure of the experimental and control groups. We adopted the inverse variance method for developing weights for individual study effects. We quantified heterogeneity using *I*^2^ and *Q* statistics.^19^ We used a random effect model to assess the population average mean difference and 95% CI of both systolic and diastolic blood pressure according to the task-sharing groups. To assess each study's contribution towards overall heterogeneity, we conducted a sensitivity analysis by subsequently adding studies and noting the change in heterogeneity. In order to measure the dispersion of the pooled effect across study settings, we generated predictions intervals.^20^ We did exploratory subgroup analyses of the length of interventions, study participant characteristics, physician density in the country, and sample size, for estimating any potential difference in the pooled average effect of intervention on blood pressure. Individual study effects and pooled effects were visualised through forest plots. Publication bias was assessed graphically through funnel plot asymmetry and statistically by Egger's regression test.^21^ Data were pooled and analysed using “meta” package of R (version 3.5.1).^22^ Quality of the evidence was evaluated using GRADE criteria.^23^

### Role of the funding source

There was no funding for this study. The corresponding author had full access to all the data in the study and had final responsibility for the decision to submit for publication.

## Results

**Figure 1:**
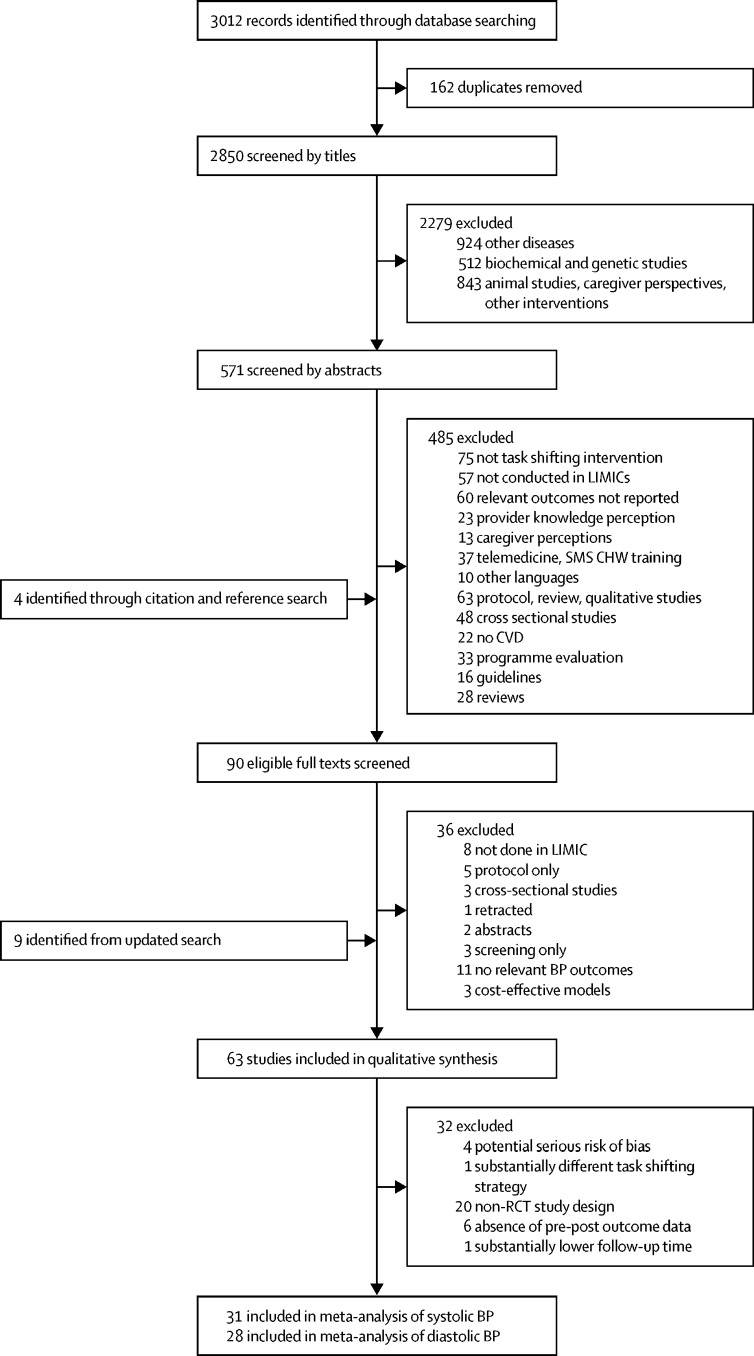
Literature search and article inclusion SMS=short messaging service. CHW=community health worker. CKD=chronic kidney disease. LMIC=low-income and middle-income country. CVD=cardiovascular disease. SBP=systolic blood pressure. DBP=diastolic blood pressure. RCT=randomised controlled trial.

We identified a total of 3012 references from our searches (figure 1). After removing 162 duplicates, we screened the titles of 2850, 2279 of which were excluded. We screened 571 abstracts and identified 86 potentially eligible articles. Another four studies were included after manually searching. The full texts of 90 studies were reviewed and 36 were excluded. Thus, we included 54 studies. An updated hand search and database search done up to December, 2018, identified an additional nine eligible studies. Thus, 63 studies were included in the narrative review and 31 studies (trials) were included in the meta-analyses.

Of the 63 studies included in the review, there were 32 randomised controlled trials (from 33 publications),^24^, ^25^, ^26^, ^27^, ^28^, ^29^, ^30^, ^31^, ^32^, ^33^, ^34^, ^35^, ^36^, ^37^, ^38^, ^39^, ^40^, ^41^, ^42^, ^43^, ^44^, ^45^, ^46^, ^47^, ^48^, ^49^, ^50^, ^51^, ^52^, ^53^, ^54^, ^55^, ^56^ 11 cluster randomised trials,^57^, ^58^, ^59^, ^60^, ^61^, ^62^, ^63^, ^64^, ^65^, ^66^, ^67^ and 20 before-and-after studies^68^, ^69^, ^70^, ^71^, ^72^, ^73^, ^74^, ^75^, ^76^, ^77^, ^78^, ^79^, ^80^, ^81^, ^82^, ^83^, ^84^, ^85^, ^86^, ^87^ (appendix pp 3–38). 50 studies were done in Asia and Africa. Studies mostly took place in the community (n=15), primary health centres (n=24), outpatient clinics (n=16), and hospitals (n=8). Interventions were delivered by nurses (n=30), pharmacists (n=10), dietitians (n=4), and community health workers (n=19). We included 31 studies with 13 489 participants in the meta-analyses. Individual study sizes ranged from 35 to 3977 participants. 16 studies lasted 12 months or more. The nature of the interventions varied. We categorised them into lifestyle modifications through education for participants, follow-up care, algorithm-based management, non-physician drug prescription, referrals, and organisation of care.

We assessed the quality of trials using the Cochrane risk of bias tool (appendix p 34). Four studies^41^, ^49^, ^51^, ^56^ did not mention randomisation methods and the randomisation method was unclear in five studies.^24^, ^25^, ^27^, ^36^, ^59^ Only 15 of 43 trials reported details of allocation concealment.^29^, ^32^, ^35^, ^36^, ^39^, ^45^, ^46^, ^47^, ^50^, ^53^, ^55^, ^63^, ^64^, ^65^, ^66^, ^67^ Two studies^29^, ^30^ reported participant blinding and 14 studies^25^, ^26^, ^29^, ^40^, ^44^, ^46^, ^50^, ^51^, ^52^, ^53^, ^55^, ^66^ reported outcome assessor blinding.

We included 43 trials and 20 before-and-after studies for narrative synthesis. Most of the trials recruited participants with hypertension (n=16), another ten recruited patients with diabetes, eight with cardiovascular disease (two of which were in patients who had had a stroke), five from the general population, two with overweight or metabolic syndrome, one with post-acute coronary syndrome, and one with dyslipidaemia. The interventions were facilitated by nurses (n=21), dietitians (n=4), pharmacists (n=7), and community health workers (n=11).

Education about lifestyle modifications was a component of the intervention in most studies (41 [95%] of 43), including education on diet (n=31), physical activity (n=25), and reducing smoking (n=13) and alcohol consumption (n=8). Two studies^26^, ^44^ began health education at hospital and continued at home, after discharge from hospital. Hacihasanoğlu and colleagues^41^ did a three group trial, with a control group who received usual care, an intervention group who received health education on medication adherence, and another group who received education about medical adherence, diet, and importance of physical activity. Two studies^42^, ^46^ used nurses trained in motivational interviewing to involve participants in identifying problems, setting goals, and creating action plans to modify health. Pharmacist-led studies included monitoring of drug-related problems (such as changes in blood pressure or side-effects) in addition to lifestyle counselling.^31^, ^39^, ^49^

Home visits were part of the intervention in ten studies.^24^, ^26^, ^41^, ^44^, ^47^, ^48^, ^53^, ^56^, ^58^, ^64^ Home visits were made to impart or reinforce lifestyle education and for home blood pressure monitoring. Two studies^52^, ^64^ used community health workers to monitor blood pressure at participants' homes, and another two^41^, ^56^ used nurses to monitor blood pressure at home. Labhardt and colleagues^36^ used reminder letters for missed appointments by participants.

Seven studies tested algorithm-based management.^38^, ^45^, ^59^, ^61^, ^62^, ^65^, ^66^ Ali and colleagues,^45^ Tian and colleagues,^62^ and Prabhakaran and colleagues^66^ implemented algorithm-based disease management by using an electronic decision support system. Four studies^59^, ^61^, ^62^, ^65^ tested a treatment algorithm and automatic decision prompt to initiate prescriptions of hypertension drugs.

Seven studies^46^, ^48^, ^54^, ^62^, ^63^, ^64^, ^65^, ^66^ tested a referral system for participants with high or uncontrolled blood pressure. In two studies,^62^, ^64^ community health workers referred patients to health-care facilities for anti-hypertensive drugs. Goudge and colleagues^63^ used community health workers to assist nurses in organising care such as booking appointments and telephoning with reminders for participants with hypertension. Ogedegbe and colleagues^65^ referred patients with high cardiovascular risk to hospital for further management.

Six of 43 randomised controlled trials did not report the outcome variable of interest (difference in blood pressure before and after the intervention). Additionally, four studies were excluded because of inadequate description of randomisation procedure, one study was excluded because it was a feasibility trial with less than 3 months of follow-up, and another one was excluded because of differences in task sharing (involving non-blood pressure drug titrations across multiple therapeutic areas). Finally, 31 trials were included in the meta-analyses (including two groups from Azami and colleagues' study^55^ and three groups from Neupane and colleagues' study^64^). In the meta-analyses only randomised controlled trials and cluster randomised controlled trials were included.

**Figure 2:**
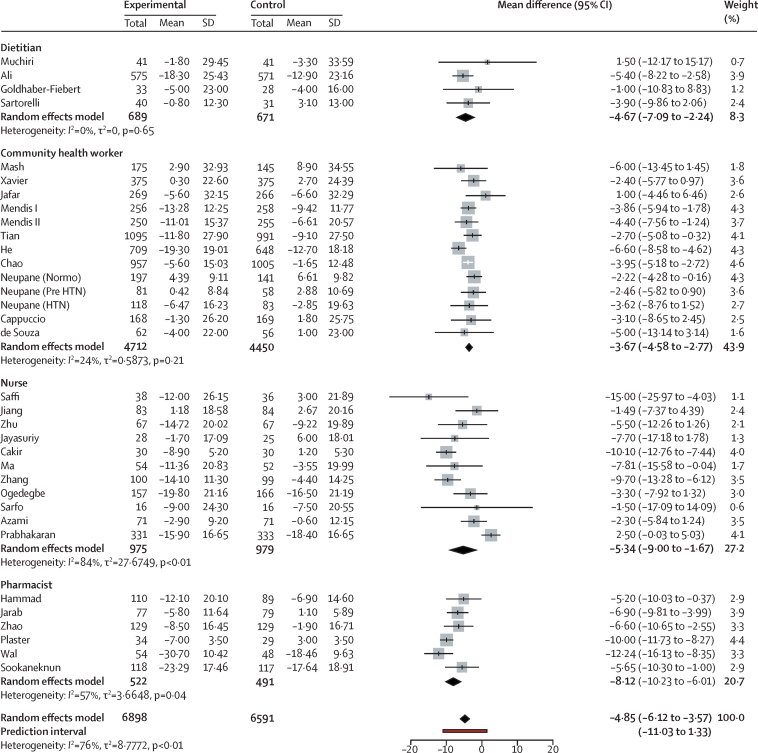
Systolic blood pressure changes with task sharing compared with usual care

**Figure 3:**
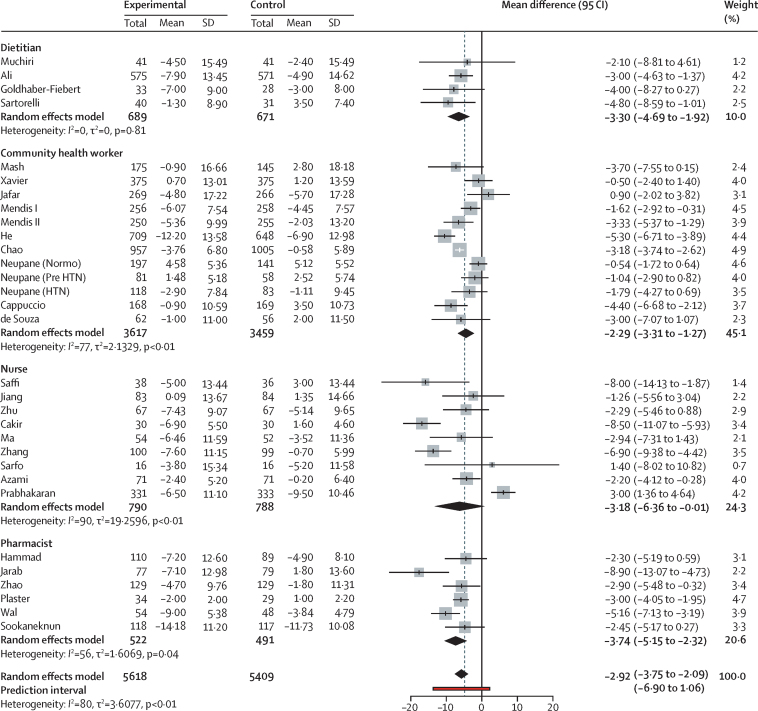
Diastolic blood pressure changes with task sharing compared with usual care

The population average mean difference between before and after measurements of systolic blood pressure ranged from −3·67 mm Hg (95% CI −4·58 to −2·77, *I*^2^=24%; for interventions delivered by community health workers) to −8·12 mm Hg (–10·23 to −6·01, *I*^2^=57%; for interventions delivered by pharmacists). Dietitians delivering the intervention resulted in an average mean difference of −4·67 mm Hg (–7·09 to −2·24; *I*^2^=0%), and when nurses delivered the intervention it was −5·34 mm Hg (–9·00 to −1·67; *I*^2^=84%; figure 2). Overall, the average mean difference in systolic blood pressure was −4·85 mm Hg (–6·12 to −3·57; *I*^2^=76%). The prediction interval for the systolic blood pressure pooled estimate was −11·03 to 1·33. Funnel plots for publication bias did not show any asymmetry (appendix p 36) and the Egger's regression test did not indicate bias (*t*=0·12, degrees of freedom=32, p value=0·90). We found a similar reduction in diastolic blood pressure (overall mean difference −2·92 mm Hg, −3·75 to −2·09, *I*^2^=80%). The average mean difference of diastolic blood pressure ranged from −2·29 to −3·74 mm Hg, depending on the task sharing group (figure 3). The prediction interval of the diastolic blood pressure pooled average estimate ranged from −6·90 to 1·06. We found no evidence of bias for diastolic blood pressure in funnel plots (appendix p 37) or from Egger's regression test (*t*=–0·365, degrees of freedom=29, p value=0·71).

Subgroup analyses demonstrated larger systolic blood pressure responses in high-risk individuals (individuals with diabetes mellitus, coronary artery disease, or hypertension) compared with interventions implemented in the general population (appendix p 38). Similar differences were also observed for the average mean difference in diastolic blood pressure, but the mean diastolic blood pressure differences were higher in coronary artery disease and diabetes populations (appendix p 39). Studies of patients with hypertension, studies of pharmacist-led interventions, and studies with smaller sample size had larger reductions in both systolic and diastolic blood pressure (appendix pp 39–43). Additionally, blood pressure response was greatest in studies with short follow-up compared with studies with medium or long follow-up (appendix pp 40–41). In general, blood pressure reductions were better in smaller trials (n<500) than in larger trials (n>500; appendix pp 42–43). Blood pressure changes were largest in settings with more physicians (appendix pp 44–45).

Due to considerable heterogeneity, we did an exploratory sensitivity analysis by using the leave-one-study-out method. The exclusion of two studies^32^, ^66^ reduced the overall *I*^2^ from 76·3% to 57·3% for systolic blood pressure. Excluding these two studies from the analysis of diastolic blood pressure caused a small reduction in overall *I*^2^ (from 79·5% to 71·3%), with little change in the population average: systolic blood pressure change was −4·92 mm Hg and diastolic blood pressure change was −3·10.

In total, 20 before-and-after design studies were included in the review and results are summarised descriptively. There were three studies each from Cameroon,^73^, ^74^, ^75^ India,^70^, ^76^, ^82^ and Thailand,^71^, ^72^, ^84^ two each from Iran,^77^, ^85^ Guatemala,^80^, ^86^ and Nigeria,^69^, ^88^ and one each from Honduras,^78^ Ghana,^83^ Mexico,^79^ and South Africa.^68^ One multicentre study was done in Bangladesh, Pakistan, and Sri Lanka.^81^ Three studies,^76^, ^77^, ^79^ recruited participants from the general population, whereas all other studies were conducted in high-risk participants such as individuals with hypertension, diabetes mellitus, and cardiovascular disease. Similar to randomised controlled trials, the intervention tasks were shared with nurses, pharmacists, health promotion specialist, and community health workers. All the included studies used life-style modification education for the participants. Group and individual life-style education were used. In one study,^86^ life-style education was delivered in a series of home visits. The content of life-style education varied from diet (n=15), physical activity (n=9), and medication adherence (n=6) among studies. 11 studies^69^, ^76^, ^77^, ^78^, ^79^, ^80^, ^81^, ^84^, ^85^, ^86^ delivered life-style education alone for managing blood pressure, eight of which reported a reduction in blood pressure following the lifestyle intervention. However, three studies^77^, ^79^, ^84^ did not show a reduction in blood pressure,^77^, ^84^ or found an increase in blood pressure among elderly participants.^79^

Most of the studies had a clear objective and eligibility criteria reported (appendix 22–28). However, whether participants were representative of the clinical population was difficult to determine because of unclear reporting of recruitment methods in nine studies. All studies described the intervention but none reported blinding of outcome assessors. Sample size calculations were reported in six studies,^70^, ^71^, ^79^, ^83^, ^84^, ^87^ statistical pre-post tests were done in 19 studies, and 14 studies^68^, ^70^, ^72^, ^73^, ^74^, ^75^, ^77^, ^78^, ^79^, ^81^, ^82^, ^84^, ^86^, ^87^ adjusted for potential confounders.

Four studies^70^, ^72^, ^80^, ^84^ had follow-up interventions for management of blood pressure. Two studies each employed nurses^72^, ^84^ and community health workers^54^, ^64^ for follow up. Navichraren and colleagues^84^ telephoned participants following life-style education classes but reported no difference in blood pressure. Other studies used home visits to impart life-style modification education. A study done in India^70^ used cardiovascular disease risk profiling and referral to physicians along with follow-up by community health workers to reinforce risk reduction and adherence to treatment. It showed a significant fall in systolic blood pressure of 8·8 mm Hg. Similarly, a study done in Guatemala^80^ had nurse-led cardiovascular risk management and follow-up by community health workers, resulting in a mean reduction of 27·2 mm Hg for systolic blood pressure and 7·7 mm Hg for diastolic blood pressure. Suwanphan and colleagues^72^ arranged for six consecutive monthly home visits, but found no statistically significant improvements in blood pressure.

All the studies using protocol-based care showed a reduction in blood pressure. In a multicountry study^81^ the overall mean systolic blood pressure decreased by 4·5 mm Hg (95% CI 2·3 to 6·7). Two studies in which community health-care workers delivered the intervention also resulted in reduction in mean systolic blood pressure (by 8·8 mm Hg in one study,^70^ 27·2 mm Hg in another^80^). In one study,^82^ nurse care coordinators used a clinical decision support system on a mobile phone to generate a prescription, which was vetted by a physician, which reduced systolic blood pressure by 14·6 mm Hg (95% CI 15·3 to 13·8) and diastolic blood pressure by 7·6 mm Hg (8·0 to 7·2). In South Africa,^68^ 68% of patients with hypertension achieved blood pressure control through a stepwise process of diagnosis, management, and appropriate referral. Although the three Cameroonian studies^73^, ^74^, ^75^ reported statistically significant reductions in mean blood pressure (of 11·7/7·8 mm Hg, 5·9/3·3 mm Hg, and 22·8/12·4 mm Hg), they had high drop-out rates. In a study done in Nigeria,^87^ pharmacy staff had regular consultations with patients for blood pressure measurements and lifestyle counselling (with the help of an mHealth application and under the supervision of a cardiologist) and achieved a mean reduction of 9·9 mm Hg (SD 18) in systolic blood pressure.

In three studies from Cameroon, nurses could prescribe medicines, which resulted in meaningful reductions in average blood pressure (11·7/7·8 mm Hg in one study,^73^ 5·9/3·3 mm Hg in another,^74^ and 22·8/12·4 mm Hg in a third^75^). In two other studies,^68^, ^80^ nurses could prescribe first-line hypertension drugs. However, in cases of newly diagnosed hypertension or in individuals with high cardiovascular risk, nurses had to seek advice from physicians before initiating treatment. Both studies showed a significant reduction in blood pressure. Ajay et al^82^ tested the effects of nurse care coordinators using a clinical decision support system on a mobile phone to generate a prescription that was vetted by a physician and showed a 14·6 mm Hg (95% CI 15·3 to −3·8) reduction in systolic blood pressure and a 7·6 mm Hg (95% CI 8·0 to 7·2) reduction in diastolic blood pressure.

Nine studies^68^, ^70^, ^74^, ^75^, ^80^, ^81^, ^82^, ^83^, ^87^ tested arrangements for long-term organised care in which a nurse or community health worker referred participants to a physician or health-care facility. Organisation of care ranged from coordination of groups for education, appointment reminders, medication dispensing, screening for cardiovascular disease risk, and referral for further treatment. However, the effectiveness of arrangements for follow-up on patient outcomes was not assessed specifically.

In terms of study quality, the evidence for task-sharing interventions to lower systolic blood pressure delivered by nurses, pharmacists, and community health workers was classed as moderate because of indirectness and inconsistency. The evidence for managing diastolic blood pressure was rated as low because of both indirectness and inconsistency. We did not downgrade studies for lack of masking because implementation of task-sharing interventions in real life is unlikely to be masked.

## Discussion

We systematically reviewed the effectiveness of task-sharing interventions for managing blood pressure in LMICs and identified studies involving nurses, dietitians, pharmacists, and community health workers. Overall, task-sharing interventions led to reduction in average blood pressure levels. Although the prediction intervals indicate variation in blood pressure responses across study settings, the interpretation of prediction intervals are compromised when study sizes vary.^88^ Our analyses indicate that the blood pressure response is lowest in settings where the density of physicians is lowest. However, in settings with higher physician density, even within LMICs, we found better blood pressure responses. Despite the moderate-to-high heterogeneity in the pooled results, the overall quality of evidence was moderate for systolic blood pressure, and low for diastolic blood pressure on GRADE criteria.

Healthy life-style education was the mainstay of most of the interventions studied. Although all types of non-physician health workers were involved in delivery of the intervention, the effectiveness was relatively better when given by workers with more education such as nurses and pharmacists. The higher order groups of workers were involved in activities such as protocol-based care, decision support systems for screening, stratification, triage, and medication adherence monitoring in addition to life-style education. A review of task-shifting by Ogedegbe and colleagues^89^ found that involving non-physician health-care workers such as nurses in prescribing of medications, treatment, or medical testing significantly improved blood pressure and glucose levels. Task-sharing interventions should be designed with an understanding of the specific health service delivery context. WHO provides a framework and global recommendations for task sharing from medical doctors to nurses and community health workers for HIV/AIDS care in low-resource settings.^7^ Nurses or pharmacists can take referrals from community health workers for basic anti-hypertensive medication prescriptions or titration of medications, thus leaving physicians to care for complex cases. The range of task-sharing strategies implemented in a study in Ghana might be an ideal example,^65^ where nurses are engaged in cardiovascular risk screening, life-style counselling, and initiation or titration of hypertension medications. A Cochrane review^90^ showed that nurse-led or pharmacist-led care could effectively improve blood pressure control. Therefore, it is essential to have policies on collaborative care models that involve non-physician prescriptions and organisation of health-care service for better task sharing in low-income and high-income countries.

All of the community health worker-led intervention studies included in our review focused on life-style education mainly at home or in community settings. Community health workers in LMICs are engaged in health promotion activities in reproductive health and family planning and they conduct regular home visits in their assigned areas. It is therefore practically possible for them to also support non-communicable disease prevention in home and community settings. Furthermore, engaging community health workers in cardiovascular screening in low-resource settings is considered as a cost-effective strategy for averting mortality.^91^ A structured group education programme^92^ delivered by mid-level trained health-care workers at community health centres in South Africa has been found to be both effective in reducing blood pressure and cost-effective.

Prior studies^91^, ^93^ have shown that community health workers with adequate training can successfully screen for blood pressure and other cardiovascular risk factors. However, screening for risk factors should be adequately supported by lifestyle education, basic prescription of drugs, and proper referral for managing complex conditions. Referrals to primary care centres were increased due to community health worker interventions in several studies.^63^, ^81^ However, these primary care centres face shortages of drugs, failure of equipment, and insufficient physician time due to increased patient load. Along with task sharing it is therefore important to increase access to medicines for better blood pressure control.

Task sharing without health system strengthening, restructuring, and health-care regulation will not yield any desirable results.^94^ Several studies^62^, ^66^, ^82^, ^87^ demonstrated that care led by nurses and pharmacists with electronic decision support systems and algorithms can manage blood pressure effectively. However, to successfully implement these measures across a health system, in-service training, supportive supervision, and expansion of job descriptions will be needed. Incorporating dietitians or nutritionists into public health systems for prevention and management of non-communicable diseases should be explored further. Additionally, future studies should evaluate the role of collaborative care in engaging different cadres of the health workforce simultaneously in prevention and control of hypertension.

Many of the interventions studied were multifaceted. In some studies, a care coordinator, aided by an electronic decision support system, acted as a link between patients and physicians for better blood pressure control and other risk factor management. This approach has a component of both mHealth and task sharing for managing multiple risk factors simultaneously. Although the relative effectiveness of the individual components has not been evaluated, there is some evidence^90^ that multiple risk factor interventions might lower blood pressure. He and colleagues^52^ and Jafar and colleagues^81^ also reported multicomponent interventions in which the strategies were cumulatively effective in reducing blood pressure. Fairall and colleagues^61^ tested task sharing for a range of conditions such as diabetes, hypertension, chronic respiratory disease, and depression. Such integrated disease management strategies are essential to improve delivery of care for chronic diseases. A systematic review^95^ investigated the effectiveness of integrated care and found that patient access to services was largely improved compared with routine care.

Although studies with long-term follow-up demonstrate the effectiveness of task sharing for reducing blood pressure, the effect was moderate as compared with short-term follow-up studies. Maintenance of healthy behaviours for the entire follow-up period might require sustained efforts. Long-term follow-up studies should also focus on evaluating the effectiveness of interventions in reducing clinically relevant patient-oriented outcomes such as mortality and quality of life.

Strengths of the review included a comprehensive search strategy in multiple databases. The inclusion of different study designs enabled us to review different types of intervention for managing blood pressure. We have incorporated studies irrespective of participants' hypertension status. Our review has limitations. We did not categorically identify the influence of each intervention method on blood pressure control. However, we analysed the treatment effects based on intervention providers, treatment, or follow-up duration. The strength of the overall quality of evidence was hampered due to potential bias resulting from unclear randomisation, allocation concealment, and blinding, and improper analysis in cluster randomised trials. We excluded the study by Fairall and colleagues^61^ because of considerable differences from the rest of the studies. It deals with task-sharing to authorise prescription of an expanded range of drugs such as enalapril and amlodipine for hypertension, glibenclamide and gliclazide for diabetes, and simvastatin for increased cardiovascular risk. Lastly, we might have missed studies not published in English.

In conclusion, task-sharing interventions for managing hypertension in LMICs show potential in reducing blood pressure. However, further implementation research is needed to understand the implications for health systems and patient-oriented outcomes. Future research should focus on ascertaining how the interventions fare in community settings. Implementation research such as nurse-led hypertension management in western Kenya^96^ might show further usefulness of task sharing. How health-care teams and systems can ensure continuity of task-sharing interventions should also be investigated. Future studies should also include information regarding the health workforce available. Assessing the cost effectiveness of task-sharing interventions, such as done in Argentina,^97^ will aid decision making. Understanding barriers and facilitators of scale up in diverse settings should also be studied. Policies to enable wider implementation of task-sharing interventions for control of blood pressure along with other risk factors for non-communicable diseases are needed.

## References

[1] Roth GA, Johnson C, Abajobir A, et al. Global, regional, and national burden of cardiovascular diseases for 10 causes, 1990 to 2015. J Am Coll Cardiol 2017; 70: 1–25. 10.1016/j.jacc.2017.04.052 PMC5491406 28527533

[2] Hay SI, Abajobir AA, Abate KH, et al. Global, regional, and national disability-adjusted life-years (DALYs) for 333 diseases and injuries and healthy life expectancy (HALE) for 195 countries and territories, 1990–2016: a systematic analysis for the Global Burden of Disease Study 2016. Lancet 2017; 390: 1260–344. 10.1016/S0140-6736(17)32130-X PMC5605707 28919118

[3] Mancia G, Fagard R, Narkiewicz K, et al. 2013 ESH/ESC Guidelines for the management of arterial hypertension: the Task Force for the management of arterial hypertension of the European Society of Hypertension (ESH) and of the European Society of Cardiology (ESC). J Hypertens 2013; 31: 1281–357. 10.1097/01.hjh.0000431740.32696.cc 23817082

[4] Irazola VE, Gutierrez L, Bloomfield G, et al. Hypertension prevalence, awareness, treatment, and control in selected LMIC communities: results from the NHLBI/UHG network of centers of excellence for chronic diseases. Global Heart 2016; 11: 47–59. 10.1016/j.gheart.2015.12.008 PMC4843831 27102022

[5] Naghavi M, Abajobir AA, Abbafati C, et al. Global, regional, and national age-sex specific mortality for 264 causes of death, 1980–2016: a systematic analysis for the Global Burden of Disease Study 2016. Lancet 2017; 390: 1151–210. 10.1016/S0140-6736(17)32152-9 PMC5605883 28919116

[6] Sibbald B, Shen J, McBride A. Changing the skill-mix of the health care workforce. J Health Serv Res Policy 2004; 9 (suppl): 28–38. 10.1258/135581904322724112 15006226

[7] WHO, PEPFAR, UNAIDS. Task shifting: rational redistribution of tasks among health workforce teams: global recommendations and guidelines. Geneva: World Health Organization, 2007.

[8] Dawson AJ, Buchan J, Duffield C, Homer CSE, Wijewardena K. Task shifting and sharing in maternal and reproductive health in low-income countries: a narrative synthesis of current evidence. Health Policy Plan 2014; 29: 396–408. 10.1093/heapol/czt026 23656700

[9] Joshi R, Thrift AG, Smith C, et al. Task-shifting for cardiovascular risk factor management: lessons from the Global Alliance for Chronic Diseases. BMJ Glob Health 2018; 3 (suppl 3): e001092. 10.1136/bmjgh-2018-001092 PMC6231102 30483414

[10] Bergström S. Training non-physician mid-level providers of care (associate clinicians) to perform caesarean sections in low-income countries. Best Pract Res Clin Obstet Gynaecol 2015; 29: 1092–101. 10.1016/j.bpobgyn.2015.03.016 25900128

[11] Lewin S, Munabi-Babigumira S, Glenton C, et al. Lay health workers in primary and community health care for maternal and child health and the management of infectious diseases. Cochrane Database Syst Rev 2010; 3: CD004015. 10.1002/14651858.CD004015.pub3 PMC6485809 20238326

[12] Callaghan M, Ford N, Schneider H. A systematic review of task-shifting for HIV treatment and care in Africa. Hum Resour Health 2010; 8: 8. 10.1186/1478-4491-8-8 PMC2873343 20356363

[13] Ogedegbe G, Plange-Rhule J, Gyamfi J, et al. A cluster-randomized trial of task shifting and blood pressure control in Ghana: study protocol. Implement Sci 2014; 9: 73. 10.1186/1748-5908-9-73 PMC4063247 24923300

[14] Glynn LG, Murphy AW, Smith SM, Schroeder K, Fahey T. Interventions used to improve control of blood pressure in patients with hypertension. Cochrane Database Syst Rev 2010; 3: CD005182. 10.1002/14651858.CD005182.pub4 PMC13248079 20238338

[15] Anand TN, Joseph LM, Geetha AV, Chowdhury J, Prabhakaran D, Jeemon P. Task-sharing interventions for cardiovascular risk reduction and lipid outcomes in low- and middle-income countries: a systematic review and meta-analysis. J Clin Lipidol 2018; 12: 626–42. 10.1016/j.jacl.2018.02.008 PMC5994347 29559305

[16] Higgins JPT, Altman DG, Gøtzsche PC, et al. The Cochrane Collaboration's tool for assessing risk of bias in randomised trials. BMJ 2011; 343: d5928. 10.1136/bmj.d5928 PMC3196245 22008217

[17] NHLBI. Quality assessment tool for before-after (pre-post) studies with no control group. National Heart, Lung, and Blood Institute: Bethesda, MD, USA; 2014.

[18] Rao JNK, Scott AJ. A simple method for the analysis of clustered binary data. Biometrics 1992; 48: 577–85. 1637980

[19] Higgins JP, Thompson SG. Quantifying heterogeneity in a meta-analysis. Stat Med 2002; 21: 1539–58. 10.1002/sim.1186 12111919

[20] Riley RD, Higgins JPT, Deeks JJ. Interpretation of random effects meta-analyses. BMJ 2011; 342: d549. 10.1136/bmj.d549 21310794

[21] Egger M, Smith GD, Schneider M, Minder C. Bias in meta-analysis detected by a simple, graphical test. BMJ 1997; 315: 629. 10.1136/bmj.315.7109.629 PMC2127453 9310563

[22] R Core Team. R: a language and environment for statistical computing. 2018. https://www.R-project.org/ (accessed March 10, 2019).

[23] Guyatt G, Oxman AD, Akl EA, et al. GRADE guidelines: 1. Introduction—GRADE evidence profiles and summary of findings tables. J Clin Epidemiol 2011; 64: 383–94. 10.1016/j.jclinepi.2010.04.026 21195583

[24] Garcia-Pena C, Thorogood M, Wonderling D, Reyes-Frausto S. Economic analysis of a pragmatic randomised trial of home visits by a nurse to elderly people with hypertension in Mexico. Salud Publica Mex 2002; 44: 14–20. 10.1590/s0036-36342002000100002 11910714

[25] Goldhaber-Fiebert JD, Goldhaber-Fiebert SN, Tristán ML, Nathan DM. Randomized controlled community-based nutrition and exercise intervention improves glycemia and cardiovascular risk factors in type 2 diabetic patients in rural Costa Rica. Diabetes Care 2003; 26: 24. 10.2337/diacare.26.1.24 12502654

[26] Jiang X, Sit JW, Wong TKS. A nurse-led cardiac rehabilitation programme improves health behaviours and cardiac physiological risk parameters: evidence from Chengdu, China. J Clin Nurs 2007; 16: 1886–97. 10.1111/j.1365-2702.2007.01838.x 17880478

[27] Sartorelli DS, Sciarra EC, Franco LJ, Cardoso MA. Beneficial effects of short-term nutritional counselling at the primary health-care level among Brazilian adults. Public Health Nutr 2007; 8: 820–25. 10.1079/phn2005737 16277797

[28] Sookaneknun P, Richards RME, Sanguansermsri J, Teerasut C. Pharmacist involvement in primary care improves hypertensive patient clinical outcomes. Ann Pharmacother 2004; 38: 2023–28. 10.1345/aph.1D605 15522983

[29] Cakir H, Pinar R. Randomized controlled trial on lifestyle modification in hypertensive patients. West J Nurs Res 2006; 28: 190–209. 10.1177/0193945905283367 16513919

[30] Hammad EA, Yasein N, Tahaineh L, Albsoul-Younes AM. A randomized controlled trial to assess pharmacist-physician collaborative practice in the management of metabolic syndrome in a university medical clinic in Jordan. J Manag Care Spec Pharm 2011; 17: 295–303. 10.18553/jmcp.2011.17.4.295 PMC10437647 21534640

[31] Plaster C, Melo D, Boldt V, et al. Reduction of cardiovascular risk in patients with metabolic syndrome in a community health center after a pharmaceutical care program of pharmacotherapy follow-up. Brazilian Journal of Pharmaceutical Sciences 2012; 48: 435–46.

[32] Selvaraj FJ, Mohamed M, Omar K, et al. The impact of a disease management program (COACH) on the attainment of better cardiovascular risk control in dyslipidaemic patients at primary care centres (The DISSEMINATE Study): a randomised controlled trial. BMC Fam Pract 2012; 13: 97. 10.1186/1471-2296-13-97 PMC3539990 23046818

[33] Chao J, Wang Y, Xu H, et al. The effect of community-based health management on the health of the elderly: a randomized controlled trial from China. BMC Health Serv Res 2012; 12: 449. 10.1186/1472-6963-12-449 PMC3537545 23217036

[34] Wal P, Wal A, Bhandari A, Pandey U, Rai A. Pharmacist involvement in the patient care improves outcome in hypertension patients. J Res Pharm Pract 2013; 2: 123–29. 10.4103/2279-042X.122386 PMC4076915 24991619

[35] Saffi MAL, Polanczyk CA, Rabelo-Silva ER. Lifestyle interventions reduce cardiovascular risk in patients with coronary artery disease: a randomized clinical trial. Eur J Cardiovasc Nurs 2013; 13: 436–43. 10.1177/1474515113505396 24021286

[36] Labhardt ND, Balo J-R, Ndam M, Manga E, Stoll B. Improved retention rates with low-cost interventions in hypertension and diabetes management in a rural African environment of nurse-led care: a cluster-randomised trial. Trop Med Int Health 2011; 16: 1276–84. 10.1111/j.1365-3156.2011.02827.x 21733046

[37] Jarab AS, Alqudah SG, Mukattash TL, Shattat G, Al-Qirim T. Randomized controlled trial of clinical pharmacy management of patients with type 2 diabetes in an outpatient diabetes clinic in Jordan. J Manag Care Spec Pharm 2012; 18: 516–26. 10.18553/jmcp.2012.18.7.516 PMC10437536 22971205

[38] DePue JD, Rosen RK, Seiden A, et al. Implementation of a culturally tailored diabetes intervention with community health workers in American Samoa. Diabetes Educ 2013; 39: 761–71. 10.1177/0145721713504630 PMC4062972 24052204

[39] Zhao PX Wang C, Qin L, et al. Effect of clinical pharmacist's pharmaceutical care intervention to control hypertensive outpatients in China. Afr J Pharm Pharmacol 2012; 6: 48–56.

[40] Muchiri JW, Gericke GJ, Rheeder P. Effect of a nutrition education programme on clinical status and dietary behaviours of adults with type 2 diabetes in a resource-limited setting in South Africa: a randomised controlled trial. Public Health Nutr 2016; 19: 142–55. 10.1017/S1368980015000956 PMC10270889 25872641

[41] Hacihasanoğlu R, Gözüm S. The effect of patient education and home monitoring on medication compliance, hypertension management, healthy lifestyle behaviours and BMI in a primary health care setting. J Clin Nurs 2011; 20: 692–705. 10.1111/j.1365-2702.2010.03534.x 21320198

[42] Ma C, Zhou Y, Zhou W, Huang C. Evaluation of the effect of motivational interviewing counselling on hypertension care. Patient Educ Couns 2014; 95: 231–37. 10.1016/j.pec.2014.01.011 24530144

[43] Zhu X, Wong FKY, Wu LH. Development and evaluation of a nurse-led hypertension management model in a community: a pilot randomized controlled trial. Int J Clin Exp Med 2014; 7: 4369–77. PMC4276214 25550956

[44] Xavier D, Gupta R, Kamath D, et al. Community health worker-based intervention for adherence to drugs and lifestyle change after acute coronary syndrome: a multicentre, open, randomised controlled trial. Lancet Diabetes Endocrinol 2016; 4: 244–53. 10.1016/S2213-8587(15)00480-5 26857999

[45] Ali MK, Singh K, Kondal D, et al. Effectiveness of a multicomponent quality improvement strategy to improve achievement of diabetes care goals: a randomized, controlled trial. Ann Intern Med 2016; 165: 399–408. 10.7326/M15-2807 PMC6561084 27398874

[46] Jayasuriya R, Pinidiyapathirage MJ, Jayawardena R, et al. Translational research for diabetes self-management in Sri Lanka: a randomized controlled trial. Prim Care Diabetes 2015; 9: 338–45. 10.1016/j.pcd.2015.01.014 25733343

[47] Adeyemo A, Tayo BO, Luke A, Ogedegbe O, Durazo-Arvizu R, Cooper RS. The Nigerian antihypertensive adherence trial: a community-based randomized trial. J Hypertens 2013; 31: 201–07. 10.1097/HJH.0b013e32835b0842 PMC3530610 23137954

[48] Zhu X, Wong FKY, Wu CLH. Development and evaluation of a nurse-led hypertension management model: A randomized controlled trial. Int J Nurs Stud 2018; 77: 171–78. 10.1016/j.ijnurstu.2017.10.006 29100199

[49] Shao H, Chen G, Zhu C, et al. Effect of pharmaceutical care on clinical outcomes of outpatients with type 2 diabetes mellitus. Patient Prefer Adherence 2017; 11: 897–903. 10.2147/PPA.S92533 PMC5428753 28507433

[50] Zhang P, Hu Y-D, Xing F-M, Li C-Z, Lan W-F, Zhang X-L. Effects of a nurse-led transitional care program on clinical outcomes, health-related knowledge, physical and mental health status among Chinese patients with coronary artery disease: a randomized controlled trial. Int J Nurs Stud 2017; 74: 34–43. 10.1016/j.ijnurstu.2017.04.004 28601691

[51] Hasandokht T, Farajzadegan Z, Siadat ZD, Paknahad Z, Rajati F. Lifestyle interventions for hypertension treatment among Iranian women in primary health-care settings: Results of a randomized controlled trial. J Res Med Sci 2015; 20: 54–61. PMC4354066 25767523

[52] He J, Irazola V, Mills KT, et al. Effect of a community health worker-led multicomponent intervention on blood pressure control in low-income patients in Argentina: a randomized clinical trial. JAMA 2017; 318: 1016–25. 10.1001/jama.2017.11358 PMC5761321 28975305

[53] de Souza CF, Dalzochio MB, Zucatti ATN, et al. Efficacy of an education course delivered to community health workers in diabetes control: a randomized clinical trial. Endocrine 2017; 57: 280–86. 10.1007/s12020-017-1352-z 28646377

[54] Wahab KW, Owolabi M, Akinyemi R, et al. Short-term pilot feasibility study of a nurse-led intervention to improve blood pressure control after stroke in Nigeria. J Neurol Sci 2017; 377: 116–20. 10.1016/j.jns.2017.04.005 28477678

[55] Azami G, Soh KL, Sazlina SG, et al. Effect of a nurse-led diabetes self-management education program on glycosylated hemoglobin among adults with type 2 diabetes. J Diabetes Res 2018; 4930157. 10.1155/2018/4930157 PMC6129337 30225268

[56] Huang Y-J, Parry M, Zeng Y, Luo Y, Yang J, He G-P. Examination of a nurse-led community-based education and coaching intervention for coronary heart disease high-risk individuals in China. Asian Nurs Res 2017; 11: 187–93. 10.1016/j.anr.2017.07.004 28991599

[57] Cappuccio FP, Kerry SM, Micah FB, Plange-Rhule J, Eastwood JB. A community programme to reduce salt intake and blood pressure in Ghana [ISRCTN88789643]. BMC Public Health 2006; 6: 13. 10.1186/1471-2458-6-13 PMC1382202 16433927

[58] Jafar TH, Hatcher J, Poulter N, et al. Community-based interventions to promote blood pressure control in a developing country: a cluster randomized trial. Ann Intern Med 2009; 151: 593–601. 10.7326/0003-4819-151-9-200911030-00004 19884620

[59] Mendis S, Johnston SC, Fan W, Oladapo O, Cameron A, Faramawi MF. Cardiovascular risk management and its impact on hypertension control in primary care in low-resource settings: a cluster-randomized trial. Bull World Health Organ 2010; 88: 412–19. 10.2471/BLT.08.062364 PMC2878142 20539854

[60] Mash RJ, Rhode H, Zwarenstein M, et al. Effectiveness of a group diabetes education programme in under-served communities in South Africa: a pragmatic cluster randomized controlled trial. Diabet Med 2014; 31: 987–93. 10.1111/dme.12475 PMC4232864 24766179

[61] Fairall LR, Folb N, Timmerman V, et al. Educational outreach with an integrated clinical tool for nurse-led non-communicable chronic disease management in primary care in South Africa: a pragmatic cluster randomised controlled trial. PLoS Med 2016; 13: 1–27. 10.1371/journal.pmed.1002178 PMC5119726 27875542

[62] Tian M, Ajay VS, Dunzhu D, et al. A cluster-randomized, controlled trial of a simplified multifaceted management program for individuals at high cardiovascular risk (SimCard trial) in rural Tibet, China, and Haryana, India. Circulation 2015; 132: 815–24. 10.1161/CIRCULATIONAHA.115.015373 PMC4558306 26187183

[63] Goudge J, Chirwa T, Eldridge S, et al. Can lay health workers support the management of hypertension? Findings of a cluster randomised trial in South Africa. BMJ Glob Health 2018; 3: e000577. 10.1136/bmjgh-2017-000577 PMC5841534 29527345

[64] Neupane D, McLachlan CS, Mishra SR, et al. Effectiveness of a lifestyle intervention led by female community health volunteers versus usual care in blood pressure reduction (COBIN): an open-label, cluster-randomised trial. Lancet Glob Health 2018; 6: e66–73. 10.1016/S2214-109X(17)30411-4 29241617

[65] Ogedegbe G, Plange-Rhule J, Gyamfi J, et al. Health insurance coverage with or without a nurse-led task shifting strategy for hypertension control: a pragmatic cluster randomized trial in Ghana. PLoS Med 2018; 15: 1–17. 10.1371/journal.pmed.1002561 PMC5929500 29715303

[66] Prabhakaran D, Jha D, Prieto-Merino D, et al. Effectiveness of an mHealth-based electronic decision support system for integrated management of chronic conditions in primary care: the mWellcare cluster-randomized controlled trial. Circulation 2018; 139: 380–91. 10.1161/CIRCULATIONAHA.118.038192 30586732

[67] Sarfo FS, Treiber F, Gebregziabher M, et al. Phone-based intervention for blood pressure control among Ghanaian stroke survivors: a pilot randomized controlled trial. Int J Stroke 2018; 1747493018816423. 10.1177/1747493018816423 30465630

[68] Coleman R, Gill G, Wilkinson D. Noncommunicable disease management in resource-poor settings: a primary care model from rural South Africa. Bull World Health Organ 1998; 76: 633–40. PMC2312489 10191559

[69] Oparah AC, Adje DU, Enato EF. Outcomes of pharmaceutical care intervention to hypertensive patients in a Nigerian community pharmacy. Int J Pharm Pract 2006; 14: 115–22.

[70] Kar SS, Thakur JS, Jain S, Kumar R. Cardiovascular disease risk management in a primary health care setting of north India. Indian Heart J 2008; 60: 19–25. 19212017

[71] Partiprajak S HS, Piaseu N, Brooten D, Nityasuddhi D. Outcomes of an advanced practice nurse-led type-2 diabetes support group. Pac Rim Int J Nurs Res Thail 2011; 15: 288–304.

[72] Suwanphan A SA, Jiamjarasrangsi W, Sangwatanaroj S, et al. Effectiveness of coronary heart disease risk factors reduction program for hypertensive patients in Thailand. Asian Biomed (Res Rev News) 2010; 3: 193–99.

[73] Kengne AP, Fezeu L, Sobngwi E, et al. Type 2 diabetes management in nurse-led primary healthcare settings in urban and rural Cameroon. Prim Care Diabetes 2009; 3: 181–88. 10.1016/j.pcd.2009.08.005 19748331

[74] Kengne AP, Awah PK, Fezeu LL, Sobngwi E, Mbanya J-C. Primary health care for hypertension by nurses in rural and urban sub-Saharan Africa. J Clin Hypertens 2009; 11: 564–72. 10.1111/j.1751-7176.2009.00165.x PMC8673012 19817937

[75] Labhardt ND, Balo J-R, Ndam M, Grimm J-J, Manga E. Task shifting to non-physician clinicians for integrated management of hypertension and diabetes in rural Cameroon: a programme assessment at two years. BMC Health Serv Res 2010; 10: 339. 10.1186/1472-6963-10-339 PMC3018451 21144064

[76] Balagopal P, Kamalamma N, Patel TG, Misra R. A community-based participatory diabetes prevention and management intervention in rural India using community health workers. Diabetes Educ 2012; 38: 822–34. 10.1177/0145721712459890 23033123

[77] Sarrafzadegan N, Kelishadi R, Sadri G, et al. Outcomes of a comprehensive healthy lifestyle program on cardiometabolic risk factors in a developing country: the Isfahan Healthy Heart Program. Arch Iran Med 2013; 16: 4–11. 23273227

[78] Reiger S, Harris JR, Chan KCG, Oqueli HL, Kohn M. A community-driven hypertension treatment group in rural Honduras. Glob Health Action 2015; 8: 28041. 10.3402/gha.v8.28041 PMC4567586 26362420

[79] Balcázar H, Fernández-Gaxiola AC, Pérez-Lizaur AB, Peyron RA, Ayala C. Improving heart healthy lifestyles among participants in a Salud para su Corazón promotores model: the Mexican pilot study, 2009–2012. Prev Chronic Dis 2015; 12: E34. 10.5888/pcd12.140292 PMC4362391 25764140

[80] Mendoza Montano C, Fort M, deRamirez M, Cruz J, Ramirez-Zea M. Evaluation of a pilot hypertension management programme for Guatemalan adults. Health Promot Int 2016; 31: 363–74. 10.1093/heapro/dau117 PMC4863869 25595280

[81] Jafar TH, Silva Ad, Naheed A, et al. Control of blood pressure and risk attenuation: a public health intervention in rural Bangladesh, Pakistan, and Sri Lanka: feasibility trial results. J Hypertens 2016; 34: 1872–81. 10.1097/HJH.0000000000001014 27488552

[82] Ajay VS, Jindal D, Roy A, et al. Development of a smartphone-enabled hypertension and diabetes mellitus management package to facilitate evidence-based care delivery in primary healthcare facilities in India: the mPower Heart Project. J Am Heart Assoc 2016; 5: e004343. 10.1161/JAHA.116.004343 PMC5210443 28003248

[83] Marfo AFA, Owusu-Daaku FT. Exploring the extended role of the community pharmacist in improving blood pressure control among hypertensive patients in a developing setting. J Pharm Policy Pract 2017; 10: 39. 10.1186/s40545-017-0127-5 PMC5740879 29299319

[84] Navicharern R AY, Thanasilp S. Effects of multifaceted nurse-coaching intervention on diabetic complications and satisfaction of persons with type 2 diabetes. J Med Assoc Thai 2009; 92: 1102–12. 19694337

[85] Kamran A, Sharifirad G, Heydari H, Sharifian E. The effect of theory based nutritional education on fat intake, weight and blood lipids. Electron Physician 2016; 8: 3333–42. 10.19082/3333 PMC5279963 28163845

[86] Flood D, Hawkins J, Rohloff P. A home-based type 2 diabetes self-management intervention in rural Guatemala. Prev Chronic Dis 2017; 14: E65. 10.5888/pcd14.170052 PMC5553353 28796597

[87] Nelissen HE, Cremers AL, Okwor TJ, et al. Pharmacy-based hypertension care employing mHealth in Lagos, Nigeria—a mixed methods feasibility study. BMC Health Serv Res 2018; 18: 934. 10.1186/s12913-018-3740-3 PMC6277995 30514376

[88] Partlett C, Riley RD. Random effects meta-analysis: coverage performance of 95% confidence and prediction intervals following REML estimation. Stat Med 2017, 36: 301–17. 10.1002/sim.7140 PMC5157768 27714841

[89] Ogedegbe G, Gyamfi J, Plange-Rhule J, et al. Task shifting interventions for cardiovascular risk reduction in low-income and middle-income countries: a systematic review of randomised controlled trials. BMJ Open 2014; 4: e005983. 10.1136/bmjopen-2014-005983 PMC4202019 25324324

[90] Uthman OA, Hartley L, Rees K, Taylor F, Ebrahim S, Clarke A. Multiple risk factor interventions for primary prevention of cardiovascular disease in low- and middle-income countries. Cochrane Database Syst Rev 2015; 8: CD011163. 10.1002/14651858.CD011163.pub2 PMC6999125 26272648

[91] Gaziano T, Abrahams-Gessel S, Surka S, et al. Cardiovascular disease screening by community health workers can be cost-effective in low-resource countries. Health Aff (Millwood) 2015; 34: 1538–45. 10.1377/hlthaff.2015.0349 PMC4795815 26355056

[92] Mash R, Kroukamp R, Gaziano T, Levitt N. Cost-effectiveness of a diabetes group education program delivered by health promoters with a guiding style in underserved communities in Cape Town, South Africa. Patient Educ Couns 2015; 98: 622–26. 10.1016/j.pec.2015.01.005 25641665

[93] Joshi R, Chow CK, Raju PK, et al. The rural Andhra Pradesh cardiovascular prevention study (RAPCAPS): a cluster randomized trial. J Am Coll Cardiol 2012; 59: 1188–96. 10.1016/j.jacc.2011.10.901 22440219

[94] Joshi R, Alim M, Kengne AP, et al. Task shifting for non-communicable disease management in low and middle income countries–a systematic review. PLoS One 2014; 9: e103754. 10.1371/journal.pone.0103754 PMC4133198 25121789

[95] Baxter S, Johnson M, Chambers D, Sutton A, Goyder E, Booth A. The effects of integrated care: a systematic review of UK and international evidence. BMC Health Serv Res 2018; 18: 350. 10.1186/s12913-018-3161-3 PMC5946491 29747651

[96] Vedanthan R, Kamano JH, Horowitz CR, et al. Nurse management of hypertension in rural western Kenya: implementation research to optimize delivery. Ann Glob Health 2014; 80: 5–12. 10.1016/j.aogh.2013.12.002 PMC4036099 24751560

[97] Augustovski F, Chaparro M, Palacios A, et al. Cost-effectiveness of a comprehensive approach for hypertension control in low-income settings in argentina: trial-based analysis of the hypertension control program in Argentina. Value Health 2018; 21: 1357–64. 10.1016/j.jval.2018.06.003 PMC6457112 30502778

