# Correction to *Lancet Glob Health* 2019; 7: e761–71

**DOI:** 10.1016/S2214-109X(19)30367-5

**Published:** 2019-08-28

*Anand TN, Joseph LM, Geetha AV, Prabhakaran D, Jeemon P. Task sharing with non-physician health-care workers for management of blood pressure in low-income and middle-income countries: a systematic review and meta-analysis.* Lancet Glob Health *2019; **7**: e761–71*—The acknowledgments section of this article should have stated that “This work was supported by the Wellcome Trust/DBT India Alliance Fellowship (grant number IA/CPHI/14/1/501497) awarded to Panniyammakal Jeemon”. This change has been made to the Article as of Aug 28, 2019.

