# Supplementary material for: Task sharing with non-physician health-care workers for management of blood pressure in low-income and middle-income countries: a systematic review and meta-analysis
Source: Lancet Glob Health. 2019 May 13;7(6):e761–71. doi: 10.1016/S2214-109X(19)30077-4 (PMC6527522; doi:10.1016/S2214-109X(19)30077-4)
Supplement: Supplementary appendix [file mmc1.pdf]

THE LANCET
Global Health

Supplementary appendix

This appendix formed part of the original submission and has been peer reviewed. We post it as supplied by the authors.

Supplement to: Anand TN, Joseph LM, Geetha AV, Prabhakaran D, Jeemon P. Task sharing with non-physician health-care workers for management of blood pressure in low-income and middle-income countries: a systematic review and meta-analysis. *Lancet Glob Health* 2019; **7:** e761–71.

Online supplement

Task sharing with non-physician healthcare workers for management of blood pressure in low and middle-income countries: A systematic review and meta-analysis

Corresponding author and address:

Panniyammakal Jeemon

Achutha Menon Centre for Health Science Studies

Sree Chitra Tirunal Institute for Medical Sciences and Technology

Trivandrum, Kerala, India, 695011

Email: pjeemon@gmail.com

Table S1: Characteristics of included trials in this review

Author, Year	Type of study	Country	Population/Disease condition	Task shifted to/shared with	Sample, (intervention/control)	Follow up	Intervention and Control Group	Relevant results
García-Peña et al ,2002	RCT	Mexico	Hypertension	Nurses	683(345/338)	6 months	Home visits by nurses who gave health and lifestyle advice to the participants. Control: Routine care by family physicians	The difference in the mean change in SBP was 3.31 mm Hg (95% CI 6.32, 0.29; p=0.03) compared with the control group. In DBP the difference was 3.67 (95% CI 5.22, 2.12; p<0.001).
Goldhaber-Fiebert et al,2002	RCT	Costa-Rica	Type II DM	Nutritionists	75	3 months	11 weekly nutrition education by nutritionists Control: Routine care	No statistically significant difference in SBP and DBP.
Jiang X et al,2003	RCT	China	Coronary heart disease	Nurses	167(83/84)	6 months	Intervention: Hospital-based patient/family education and Home-based cardiac rehabilitation. Control: Routine care	Intervention group had better medication adherence and significantly better control of SBP and DBP at 3 months.
Sartorelli et al , 2005	RCT	Brazil	High-risk group (overweight and relatives of DM patients)	Nutritionist	104,(83/21)	12 months	Dietary counselling which was individualized Control: Routine care	Follow up at 12 month showed an increase of DBP in the control group.
Sookaneknun et al,2004	RCT	Thailand	Hypertension	Pharmacist	235 (118/117)	6 months	Pharmacist provided life style modification education,	The treatment group had a significant

							identification of drug related problems and adherence management Control: Routine care	reduction in SBP and DBP compared with control group (p = 0.037, 0.027, respectively).
Cakir H et al,2006	RCT	Turkey	Hypertension	Nurse	60(32/38)	6 months	Comprehensive life style modification education Control: Routine care	From baseline to 6months,the mean reduction in SBP and DBP were 8.8 (SD = 5.2) and 6.9 (SD = 5.3) mmHg, respectively
Hammad et al, 2009	RCT	Jordan	Metabolic syndrome	Pharmacist	199(110/89)	6 months	Pharmacist recommendations and care counselling for the intervention group Control: Routine care	From baseline to 6months, the reduction in SBP was 12.1 (20.1) mm Hg in the intervention group vs 6.9 (14.6) mm Hg in the usual care group (P = 0.018). The reduction in DBP was 7.2 (12.6) mm Hg in the intervention group vs 4.9 (8.1) mm Hg in the usual care group (P = 0.049).
Plaster C P et al, 2009	RCT	Brazil	Metabolic syndrome	Pharmacist	120(36/38)	6 months	Intervention: Pharmacist physician collaborative care	20.7% patients in the intervention group were adherent to

							Control: Conventional treatment	medications and had CVD risk reduction.
Selvaraj et al, 2009	RCT	Malaysia	Dyslipidemia	Nurse Educators	297	12 months	Intervention: Physician and Nurse educator -COACH Programme received bi-weekly telephone follow-up by trained nurse educators and reinforcement for medication adherence.<br>Control- PCP COACH Programme only received care from the site investigators as per normal practice.	No significant difference between groups in statin adherence, SBP and DBP at the end of 12 months.
Chao J, et al, 2009	RCT	China	Elderly population	Community health centre staff, managers and researchers	1962(957/1005)	18 months	Health management programme with health education on diet, exercise program, telephone consultation and access to health promotion materials	Intervention group demonstrated improvements in regular BP monitoring and SBP.
Wal P et al,2010	RCT	India	Hypertension	Pharmacist	54	6 months	Pharmacist led care with health education on diet, exercise, smoking and alcohol. Additionally information regarding anti-hypertensive	The difference between blood pressure readings from the baseline to the second follow-up was significant for

							medication was given.	systolic [(P = 0.0001), 12.24 mmHg] and diastolic BP [(P = 0.001), 5.17 mmHg] in the intervention group.
Saffi et al,2010	RCT	Brazil	Coronary heart diseases	Nurses	74(38/36)	12 months	Intervention: Individual counselling sessions and telephone follow up with the recruiting nurse for management of cardiovascular risks and lifestyle parameters.<br>Control: usual care	SBP, and DBP were statistically significant at the end of one year.
Labhardt et al,2011	RCT	Cameroon	CVD	Nurses	221	12 months	Intervention: Group 2 received the incentive of 1 month of free treatment every month of regularly respected visits, and group 3 received reminder letters in case of a missed follow-up visit. Control: Group 1-Usual care	Overall average systolic BP decreased from 175.8 to 135.6 mmHg (95% CI: 35.0–45.4, $P < 0.001$) and diastolic from 100.7 to 80.1 (17.3–23.9; $P < 0.001$).
Jarab et al,2011	RCT	Jordan	Type II DM	Pharmacist	171(85/86)	6 months	Intervention: a comprehensive clinical pharmacy	The intervention group compared with the usual care

							service consisting of patient education on type 2 diabetes, prescription therapy, and medication adherence. Control: Conventional treatment	group had small but statistically significant improvements in the secondary measures of systolic and diastolic blood pressure, and self-reported medication adherence.
DePue et al,2010	RCT	American Samoa	Type II DM	Nurse-CHW	268(104/164)	12 months	Intervention: Evidence-based nurse-CHW team algorithm based intervention that supported diabetes self-management in a primary care setting. Control: usual care	The intervention group compared with the usual care group had small but statistically significant improvements in the secondary measures of systolic and diastolic blood pressure, and self-reported medication adherence. There were no significant differences in blood pressure, weight, or waist circumference at 12 months between groups

Zhao PX et al,2010	RCT	China	Hypertension	Pharmacist	278	6 months	To improve antihypertensive medication adherence and BP control	Significant BP controlled patients more in IG (76.4%) than in CG (50.6%) (P = 0.0000). Significant lower SBP (-8.5 mmHg, P = 0.0001) and DBP (-4.7 mmHg, P = 0.0013) levels were observed in IG.
Muchiri et al,2011	RCT	South Africa	Type II DM	Dietitian	82(41/41)	12 months	Intervention: The NE programme consisted of three components: (i) the curriculum (ii) follow-up sessions and (iii) vegetable gardening. Control: Group participants received education materials (pamphlet and wall/fridge poster) and continued with the usual medical care at their respective CHC.	No significant group differences in BMI, lipid profile, blood pressure and intakes of macronutrients, vegetables and fruits were observed.
Hacihasanog˘lu et al,2011	RCT	Turkey	Hypertension	Nurses	120	6 months	Intervention: Health education and Telephone follow up and home visits. Medication	In Groups A and B, the number of patients who regularly use medication was

							adherence and education for healthy lifestyle behaviours Control group: Routinely monitored in health care facilities.	significantly increased after education (p < 0.001); there was no significant increase in medication compliance in the control group (p > 0.05)
Ma et al,2012	RCT	China	Hypertension	Nurses	120		Intervention: Nurse led motivational counselling for hypertension management.	Mean adherence score were improved in the intervention group (p<0.05), along with SBP and DBP showed significant decrease (p<0.05).
Zhu et al,2012	RCT	China	Hypertension	Nurses	73	2 months	Intervention: Nurse-led hypertension management model. Home visits and telephone follow up Control: traditional doctor-led model	Participants from the study group led by nurses had significant improvement in self-care adherence, patient satisfaction post-intervention than those from the control group led by doctors.
Xavier et al,2012	RCT	India	Acute Coronary Syndrome (ACS)	CHW	805(404/401)	12 months	Intervention: Community health worker-based intervention for	At one year, overall adherence (≥80%) to prescribed

							adherence to drugs and lifestyle change after acute coronary syndrome. Four in-hospital and two home visits for medication adherence Control: usual care	evidence-based drugs was higher in the intervention group than in the control group (97% vs 92%, odds ratio [OR] 2·62, 95% CI 1·32–5·19; p=0·006).
Ali et al,2012	RCT	India and Pakistan	Type II DM	Non-physician care co-ordinators with training in allied health fields (such as dietetics or social work)	1146(575/571)	24 months	Intervention: Multicomponent QI strategy comprising non-physician care coordinators and decision-support electronic health records.<br>Control: usual care	Compared with usual care, intervention participants achieved larger reductions in SBP (-4.04 mm Hg [CI,-5.85 to- 2.22 mm Hg]), DBP (-2.03 mm Hg [CI,-3.00 to-1.05 mm Hg]).
Jayasuriya et al,2013	RCT	Sri Lanka	Type II DM	Nurse	85(43/42)	6 months	Diabetes self-management intervention delivered by nurses.	SBP decreased in the intervention group but not significantly.
Adeyemo et al,2013	RCT	Nigeria	Hypertension	Nurses	698	6 months	Intervention: Nurse led hypertension management and follow up home visits. Clinic-based treatment administered by trained nurses; provision of free antihypertensive	12 to 27% of patients with newly diagnosed hypertension would default from treatment in the first six months.

							medications; and provision of funds to reimburse participants for transportation costs. Titration was left to the discretion of the physicians. Control: Nurse led clinic care	
Zhu et al& ,2013	RCT	China	Hypertension	Nurses	134	3 months	Team based care. Nurses used home visits, telephone follow up and organisation of referrals for hypertension management	The mean reduction of systolic/diastolic blood pressure in the intervention and control groups was 14.37/7.43 mmHg and 5.10/2.69 mmHg, respectively ($p < 0.01$). Also, study group had significantly greater improvement in self-care behaviors such as adherence to medication than those in the control group ($p < 0.01$)
Shao H et al, 2017	RCT	China	Type II DM	Pharmacist	199(100/99)	6 months	Pharmacist led health education, face to face and telephone interview.	BP values of intervention group were slightly higher than that of

								control group for baseline, but both systolic blood pressure (SBP)and diastolic blood pressure (DBP) decreased significantly (P,0.05) after intervention.
Zhang et al, 2014	RCT	China	CAD	Nurses	199 (99/100)	7 months	Nurse led transitional care for CAD patients, with lifestyle education, home visits and telephone follow up.	Clinical outcomes showed significant differences between the control and intervention groups (SBP, t = 5.762, P = 0.000), (DBP, t = 4.250, P =0.000).
Hasandokht et al ,2014	RCT	Iran	High BP	Nurses	161(80/81)	7 months	Nurse led lifestyle education	The mean systolic BP changed from 158.8 (±8.1) mmHg to 153.2 (±6.4) mmHg during 4-week and to 145.5 (±4.6)) mmHg after 6 months in the intervention group (P < 0.001).
He J et al, 2016	RCT	Argentina	Uncontrolled hypertension	CHW	1432	18 months	Community health worker (CHW)-led multicomponent	Systolic BP reduction from baseline to month

							intervention to improve blood pressure (BP) control among low-income patients with hypertension	18 was 19.3 mmHg (95% confidence interval [CI]: 17.9, 20.8) in the intervention group and 12.7 mmHg (95% CI: 11.3, 14.2) in the control group; difference in the reduction was 6.6 mmHg (95% CI: 4.6, 8.6; p<0.001). Diastolic BP decreased by 12.2 mmHg (95% CI: 11.2, 13.2) in the intervention group and 6.9 mmHg (95% CI: 5.9, 7.8) in the control group; difference in the reduction was 5.4 mmHg (95% CI: 4.0, 6.8; p<0.001).
Sarfo et al, 2018	RCT	Nigeria	Stroke survivors with uncontrolled BP	Nurse	60(30/30)	9 months	Nurse navigators allowed for more frequent access to care for patients who use a Blue-toothed BP device and smartphone with an App. Nurse	At month 9, proportion on the intervention versus controls with BP < 140/90 mmHg was 14/30 (46.7%) versus 12/30

							navigators directly engaged participants who breached set targets and provided advice on management. Control group received usual care with study received SMS messages dealing with healthy lifestyle behaviors but not with medication adherence.	(40.0%), p = 0.79 by intention-to-treat; systolic BP < 140 mmHg was 22/30 (73.3%) versus 13/30 (43.3%), p = 0.035
de Souza et al, 2017	RCT	Brazil	TII DM	Community health workers	118(62/56)	3 months	Intervention group (CHW n = 4) received training on structured diabetes education and were taught to transmit their knowledge during their visits. Control group (CHW n = 4) received training in health issues not related to DM.	No statistical significant difference was observed between the groups for blood pressure outcome.
Wahab et al, 2017	RCT	Nigeria	Stroke	Nurse	35(17/18)	14 days	Intervention group had nurse-led group clinics which were held at the outpatient facilities of the participating hospitals and focused on education and	There was no significant difference between the baseline and post-intervention systolic blood pressure of the

							skill-building.. Patients in this study arm received usual care as determined by their providers. Usual care after stroke in Nigeria typically involves at least one scheduled follow-up visit after the initial diagnostic encounter with a neurologist, with subsequent care by a neurologist or primary care physician based on need.	intervention (138.44 ± 29.01 mmHg versus 137.50 ± 23.05 mmHg, p = 0.84) and control (138.11 ± 19.56 mmHg versus 133.14 ± 18.24 mmHg, p = 0.27) groups. Similarly, there was no significant difference between the baseline and post-intervention diastolic blood pressure of the intervention (84.56 ± 14.20 mmHg versus 84.06 ± 9.67, p = 0.90) and controls groups (85.44 ± 10.89 mmHg versus 84.17 ± 13.12 mmHg, p = 0.55)
Azami et al, 2018	RCT	Iran	T II DM	Nurse	142(71/71)	6months	Intervention group had a 12-week nurse-led diabetes management education and	Patients in the intervention group showed significant improvement in HbA1c, blood

							received follow-up telephone calls weekly. The usual diabetes care is based on the Iranian Ministry of Health Guideline on the management of the T2DM, which involves self-care management, lifestyle modification, and medication adherence.	pressure, body weight, efficacy expectation, outcome expectation, and diabetes self-management behaviours.
Huang YJ et al , 2017	RCT	China	High risk for CHD	Nurse	120(60/60)	6 months	Intervention group had group health education in the first three months and followed by a coaching support intervention, consisting of a monthly follow-up home visit and two telephone calls in the next three months. Control group had routine care provided by the community health centre and telephone follow-up as required.	The SBP of participants in the intervention group decreased by 5 mmHg ($p < .001$) and there were no changes in SBP of participants in the usual care group ($p = .169$). Compared with the usual care group, the intervention group decreased 5 mmHg more on SBP ($t = 2.01$, $p = .047$).
Cappucio FP et al, 2002	cRCT	Ghana	General population	CHW	2743	6 months	Health promotion to reduce salt intake	The IG showed a reduction in

							and improve blood pressure	SBP(2.54mmHg(-1.45 to 6.54) and DBP (3.95mmHg(0.78 to7.11) p<0.01
Jafar et al, 2009	cRCT	Pakistan	Hypertension	Lay Health workers	1341(GP+HHE n=332/HHE n=348/GP only n=335/control n=326)	24 months	Intervention: Family-based home health education (HHE) from lay health workers and annual training of general practitioners (GPs) in hypertension management. HHE alone, GP alone, HHE and GP and no intervention were the 3 arms.	The age, sex, and baseline blood pressure–adjusted decrease in systolic blood pressure was significantly greater in the HHE and GP group (10.8 mm Hg [95% CI, 8.9 to 12.8 mm Hg]) than in the GP alone.
Mendis et al, 2010	cRCT	China and Nigeria	Hypertension	Non-physician healthcare workers	2347	12 month	Intervention: Protocol based WHO CVD risk management including drug prescription. Site A-China, Site B-Nigeria Control: Conventional treatment	Systolic blood pressure at 12-month follow-up was lower compared to baseline in all groups, but reductions were greater in intervention patients than in controls in both site A (P < 0.0001) and site B (P = 0.0002). Results were

								similar for diastolic blood pressure.
Mash R J et al,2011	cRCT	South Africa	Type II DM	Health promoter	1570	12 months	Intervention: Group diabetes education led by a health promoter.<br>Control: usual care	A significant reduction in mean systolic (-4.65 mmHg, 95% CI 9.18 to -0.12; P = 0.04) and diastolic blood pressure (-3.30 mmHg, 95% CI -5.35 to -1.26; P = 0.002) in participants attending a minimum of 4 sessions.
Fairall et al, 2011	cRCT	South Africa	NCD-Hypertension	Nurses	3977	14 months	This study evaluated the impact, both benefits and harms, of introducing the expanded programme, called Primary Care 101 (PC101), in terms of the quality of primary care for four common chronic diseases: hypertension, diabetes, chronic respiratory disease, and depression.	There was poor control of hypertension and diabetes despite treatment: blood pressure was >140/90 mm Hg in 59% of hypertensive patients. It showed no benefits nor harm to the patients.
Tian et al,2014	cRCT	Rural Tibet,	CVD	CHWs	2086(1095/991)	12 months	Intervention: CHWs managed CVD	Compared with the control group,

		China, and Haryana, India.					through electronic decision support system by two medication use and two lifestyle modifications. In China CHWs were permitted to prescribe medications while in India prescriptions were authorised by physicians for aiding CHWs.	the intervention group had a 25.5% (P<0.001) higher net increase in the primary outcome of the proportion of patient-reported anti-hypertensive medication use pre-and post-intervention. There were also significant differences in certain secondary outcomes: aspirin use (net difference 17.1%, P<0.001) and systolic blood pressure (-2.7 mmHg, P=0.04)
Goudge et al,2015	cRCT	South Africa	General population	CHW	2508	18 months	CHWs to support nurses in booking appointments, filing patient records and health education on life style modification and adherence	There was no improvement in BP control among users of intervention clinics as compared with control clinics. However, the CHWs improved clinic functioning, including overall attendance, and

								attendance on the correct day.
Neupane D et al, 2015	cRCT	Nepal	General population	FCHV (female community health volunteers)	1638	12 months	Provided home visits every 4 months for lifestyle counselling and blood pressure monitoring.	The mean systolic blood pressure at 1 year was significantly lower in the intervention group than in the control group for all cohorts: the difference was –2·28 mm Hg (95% CI –3·77 to –0·79, p=0·003) for participants who were normotensive, –3·08 mm Hg (–5·58 to –0·59, p=0·015) for participants who were pre-hypertensive, and –4·90 mm Hg (–7·78 to –2·00, p=0·001) for participants who were hypertensive.
Ogedegbe G et al, 2018	cRCT	Ghana	Uncontrolled hypertension	Nurse	757(368/389)	12 months	Intervention group had health insurance coverage and scheduled nurse visits. Control group had health insurance	Intervention group with task shifting had a greater SBP reduction (−20.4 mm Hg; 95% CI −25.2 to −15.6)

							coverage for 12 months and supplemented by scheduled nurse visits.	than the health insurance group (−16.8 mm Hg; 95% CI −19.2 to −15.6. Blood pressure control improved significantly in both groups (55.2%, 95% CI 50.0% to 60.3%, for the task shifting plus health insurance group versus 49.9%, 95% CI 44.9% to 54.9%, for the health insurance group)
Prabhakaran et al,2018	cRCT	India	Hypertension and Diabetes Mellitus	Nurse	3324(1842/1856)	12 months	Using an mhealth tool nurses managed hypertension, diabetes and alcohol and tobacco use.	There was no evidence of difference between the 2 arms for systolic blood pressure (Δ=−0.98; 95% CI, −4.64 to 2.67) and glycated hemoglobin (Δ=0.11; 95% CI, −0.24 to 0.45)

&-Zhu (2014) pilot study and Zhu (2018) full study, Zhu (2018) included in the meta-analysis.

RCT= Randomised controlled trial, cRCT=Cluster randomised trial, BP=Blood pressure, SBP=Systolic Blood Pressure, DBP=Diastolic Blood Pressure, CHW=Community Health Worker, COACH=Counselling and advisory care for health, CVD=Cardiovascular Disease, PCP=Primary care physician, CAD= Coronary Artery Disease, GP= general physician, SD=standard deviation

Table S2. Characteristics of included pre-post studies in this review

Study, yr	Country	Sample size	Task shared by	Population	Characteristics of intervention	Relevant Results
Coleman, 1998	South Africa	1343	Nurses	NCD (Hypertension and Diabetes)	Development of WHO management protocols for NCD.	68% hypertension control by nurses, 82% DM control and improved adherence to medicines in hospital referred pts.
Oparah, 2006	Nigeria	36	Pharmacist	Hypertension	Pharmacist-managed hypertension clinic.	Changes in mean SBP at baseline (187.67 ± 29.46 mmHg) and at the end of the study (137.22 ± 21.65 mmHg) were significant, *P*< 0.0001. Changes in mean diastolic blood pressure at baseline (117.56 ± 21.65) and

						end of study (89 ± 17.23), were also significant (*P*< 0.0001).
Kar S S, 2008	India	1010	CHW	CVD	WHO CVD Risk assessment package used in primary health care centres.	Significant decrease in mean SBP (8.8 mm Hg) was observed during follow-up.
Partiprajak, 2008	Thailand	100	Advanced Practice Nurse(APN)	Type II DM	APN led diabetes support group vs usual care	The APN led support group members had lower SBP ($p<.05$), to those in comparison group.
Suwanphan, 2008	Thailand	781	Nurses	Hypertension	Patient life style modification education by nurses. Monthly home visit for follow up	No significant difference between two groups in terms of blood pressure.
Kengne AP,2009	Cameroon	225	Nurses	Hypertension and Diabetes	Protocol based hypertension and diabetes management including drug prescription	Blood pressure decreased significantly for systolic and marginally for diastolic blood pressure (BP decreased by 5.9/3.3 mmHg)
Kengne , 2009	Cameroon	454	Nurses	Hypertension	Nurses followed a clinical management algorithm to assist BP evaluation and drug prescription	Between baseline and final visits, systolic and diastolic blood pressures dropped by 11.7 mm Hg (95% confidence interval, 8.9–14.4) and 7.8 (95%

					and health education.	confidence interval, 5.9–9.6), respectively (P<.001).
Labhardt, 2010	Cameroon	709	Non-Physician clinicians	Hypertension and diabetes	Integrating care for hypertension and type 2 diabetes by task shifting to non-physician clinician (NPC) facilities.	Trained NPCs initiated treatment for 796 patients with hypertension and/or diabetes in two years. Among hypertensive patients with ≥ 2 documented visits (n = 493), systolic BP decreased by 22.8 mmHg (95% CI: -20.6 to -24.9; p < 0.0001) and diastolic BP by 12.4 mmHg (-10.9 to -13.9; p < 0.0001).
Balagopal,2012	India	1638	CHWs	General population	Engaging trained community health workers as change agents to provide lifestyle education	The intervention significantly reduced systolic and diastolic blood pressure by 8 mm Hg and 4 mm Hg, respectively, in the overall population.
Sarrafzadegan, 2013	Iran	2,180,000	Health workers	General Population	Multiple interventional activities were performed based on the four main strategies of healthy nutrition, increased physical activity, tobacco control and coping with stress. Of which one was	Although the mean SBP declined significantly in the intervention area versus reference area in both sexes, however the mean DBP level decreased significantly in the reference area compared to the intervention area (p < 0.0001)

					organizations and Volunteers Project<br><br>Training health workers in cities and villages;	
Reiger, 2015	Honduras	86	CHW	Hypertension	Protocol based hypertension group treatment by physician and organisation and health education by CHWs.	Hypertension control (BP140/90 mmHg), from 31.4 to 54.7% (p<0.01) after 30 months of follow up
Balcázar,<br><br>2009	Mexico	452	Promotores<br><br>(CHW)	High risk impoverished population	Cardiovascular health promotion and disease prevention activities such as health education for 12 weeks by promotores.	Post-test mean SBP among adults older than 60 years was higher than among those aged 60 years or younger (140.9 mm Hg and 120.2 mm Hg; P = .02). Among those older than 60 years, the pre- to post-test showed an increase for the mean systolic blood pressure (125.9 to 140.9; P = .04).
Montana,<br><br>2015	Guatemala	78	Nurses and CHWs	Hypertension	Nurse led hypertension management programme with health education delivered by CHWs.	The mean SBP and DBP measured at 6 months were 132.8 (SD: 15.1) and 83.4 (SD: 6.4) mmHg, showing a significant reduction of 27.2 and 7.7 mmHg of SBP and DBP from the mean baseline level (p < 0.0001).

Jafar,2016	Bangladesh<br>Pakistan<br>Sri Lanka	412	CHWs	Hypertension	Multicomponent intervention (MCI), including BP screening and home health education by trained government community health worker (CHW); providers trained in hypertension management, and compensation of CHW for additional services.	The mean SBP declined significantly by 4.5 mmHg 95% confidence interval (2.3, 6.7) mmHg ($P < 0.001$) in the overall pooled analysis in three countries; however, it varied among countries. BP decline was 10.5 mmHg (8.1, 13.0 mmHg) ($P < 0.001$) in the pooled analysis of individuals with uncontrolled hypertension at baseline, and was also significant each of the three countries.
Vamadevan ,<br>2016	India	6797	Nurse care coordinators	Hypertension and DM	Hypertension and diabetes mellitus by task-sharing with the use of a mobile phone–based clinical decision support system at community health centres	The changes in systolic blood pressure, diastolic blood pressure, and FPG observed at 18 months of follow-up were 14.6 mm Hg (95% CI: 15.3, 13.8), 7.6 mm Hg (CI: 8.0, 7.2), and 50.0 mg/dL (95% CI: 54.6, 45.5), respectively, and were statistically significant

Marfo, 2017	Ghana	180	Pharmacist	Hypertension	Community pharmacist reviewed monthly medicines use, gave health education and adherence counselling.	The mean diastolic blood pressure difference between the intervention group and the control group was statistically significant (p=0.001). The mean adherence difference between the two groups was also statistically significant at the end of the study. (p=0.001).
Navicharern<br>, 2009	Thailand	40	Nurse	Diabetes	Nurse coaching vs routine care	There was no statistically significant difference in blood pressure between the experimental group and the control group.
Kamran,<br>2016	Iran	138	Health promotion specialist	Individuals with hypertension	Nutritional advice by health promotion specialist vs routine care with instructional booklets	2 and 6 months after the intervention, systolic blood pressure in the experimental group statistically decreased (11.3 and 13 mm Hg, respectively)
Flood<br>2017	Guatemala	90	Auxiliary nurse	T2DM	Home-based diabetes education intervention.	Systolic blood pressure also improved significantly at 12 months (−6.2 mm Hg; 95% CI, −10.1 to −2.2 mm Hg; P = .002); changes in diastolic blood pressure were not significant (−1.6 mm Hg; 95% CI, −3.9 to −0.7 mm Hg; P = .17).
Nelissen<br>2018	Nigeria	102	Pharmacist	Hypertension	The key component of the	Mean systolic blood pressure decreased 9.9 mmHg (SD: 18).

					model was task-shifting from medical doctors to pharmacy staff by using a mobile application.	Blood pressure on target increased from 24 to 56% and an additional 10% had an improved blood pressure at end line, however this was not associated with duration of mHealth activity.

Table S3. Quality of pre-post studies

Study, yr	Selection bias	Observer bias (Blinding)	Measurement bias	sample size	Confounders	Causality (sufficient timeframe)
Coleman et al, 1998	Yes	CD	No	yes	No	
Oparah et al, 2006	No	CD	No	no	No	6 month
Kar S S et al, 2008	No	CD	No	yes	Yes	5 month
Partiprajak et al, 2008	No	CD	No	No	yes	NR(not reported)
Suwanphan et al 2008	No	CD	No	No	no	12 months
Kengne AP et al 2009	No	CD	No	No	no	
Kengne et al, 2009	CD, *attrition	CD	No	No	No	
Labhardt et al, 2010	No, *attrition	CD	No	No	No	2years
Balagopal et al, 2012	No	CD	No	No	No	
Sarrafzadegan et al 2013	No, *attrition	CD		Yes	Yes	5years
Reiger et al 2015	Yes, *attrition	CD	No	No	No	30 months
Balcázar et al, 2009	No	CD	No	No	yes	12 weeks
Montana et al, 2015	Yes	CD	No	No	No	6 months
Jafar et al, 2016	Yes	CD	No	No	No	3 months
Vamadevan et al, 2016	No	CD	No	No	No	18 months
Marfo et al, 2017	Yes	CD	No	yes	Yes	6 months
Navichraren et al ,2009	Yes	CD	No	yes	yes	6 months
Kamran et al,2016	No	CD	CD	no	No	6months
Flood et al, 2017	No	CD	No	No	Yes	12 months
Nelissen et al,2018	No	CD	CD	yes	Yes	6 months

No-There is no probability

Yes-There is a probability

CD- Cannot Determine

NR- Not reported

Table S4. GRADE Summary of findings

Summary of findings:

Task sharing intervention compared to usual care for managing blood pressure

Patient or population: People with primary prevention for hypertension

Setting: Low and Middle Income countries

Intervention: Task sharing intervention

Comparison: Usual care

Outcomes	Anticipated absolute effects* (95% CI)		Relative effect (95% CI)	№ of participants (studies)	Certainty of the evidence (GRADE)	Comments
	Risk with usual care	Risk with Task sharing intervention				
Systolic blood pressure change from baseline (Task shared with nurses) assessed with: mm Hg	The mean systolic blood pressure change from baseline -4.30 mm of Hg	The mean systolic blood pressure change from baseline in the intervention group was **5.34 mmHg** lower (9.00 to 1.67 lower)	-	1954 (11RCTs)	⊕⊕⊕◯ MODERATE[b]	
Diastolic blood pressure change from baseline (Task shared with nurses) assessed with: mm Hg	The mean diastolic blood pressure change from baseline -2.03 mm of Hg	The mean diastolic blood pressure change from baseline in the intervention group was **3.18 mm Hg lower** (6.36 to 0.01 lower)	-	1578 (9 RCTs)	⊕⊕◯◯ LOW[b,c]	
Systolic blood pressure change from baseline (Task shared with pharmacist) assessed with: mmHg	The mean systolic blood pressure change from baseline -6.8 mm of Hg	The mean systolic blood pressure change from baseline in the intervention group was - **8.12 mm Hg lower** (10.23 to 6.01 lower)	-	1013 (6 RCTs)	⊕⊕⊕◯ MODERATE[c]	
Diastolic blood pressure change from baseline (Task shared with pharmacist) assessed with: mm Hg	The mean diastolic blood pressure change from baseline -3.24 mm of Hg	The mean diastolic blood pressure change from baseline in the intervention group was - **3.22 mm Hg lower** (3.99 to 2.45 lower)	-	1013 (6 RCTs)	⊕⊕⊕◯ MODERATE[c]	

Systolic blood pressure change from baseline (Task shared with Community Health Workers) assessed with: mm Hg	The mean systolic blood pressure change from baseline -1.92 mm of Hg	The mean systolic blood pressure change from baseline in the intervention group was **3.67mmHg lower** (4.58 to 2.77 lower)	-	9162 (13 RCTs)	⨁⨁⨁◯ MODERATE[d]
Diastolic blood pressure change from baseline (Task shared with community health workers) assessed with: mm Hg	The mean diastolic blood pressure change from baseline -0.30 mm of Hg	The mean diastolic blood pressure change from baseline in the intervention group was **2.29 mm Hg** lower (3.31 to 1.27 lower)	-	7076 (12 RCTs)	⨁⨁◯◯ LOW[c,d]

***The risk in the intervention group** (and its 95% confidence interval) is based on the assumed risk in the comparison group and the **relative effect** of the intervention (and its 95% CI).
CI: Confidence interval; **MD:** Mean difference

GRADE Working Group grades of evidence
High certainty: We are very confident that the true effect lies close to that of the estimate of the effect
Moderate certainty: We are moderately confident in the effect estimate: The true effect is likely to be close to the estimate of the effect, but there is a possibility that it is substantially different
Low certainty: Our confidence in the effect estimate is limited: The true effect may be substantially different from the estimate of the effect
Very low certainty: We have very little confidence in the effect estimate: The true effect is likely to be substantially different from the estimate of effect

Table S5: PRISMA Checklist

Section/topic	#	Checklist item	Reported on page #
**TITLE**			
Title	1	Identify the report as a systematic review, meta-analysis, or both.	1
**ABSTRACT**			
Structured summary	2	Provide a structured summary including, as applicable: background; objectives; data sources; study eligibility criteria, participants, and interventions; study appraisal and synthesis methods; results; limitations; conclusions and implications of key findings; systematic review registration number.	2
**INTRODUCTION**			
Rationale	3	Describe the rationale for the review in the context of what is already known.	3-4
Objectives	4	Provide an explicit statement of questions being addressed with reference to participants, interventions, comparisons, outcomes, and study design (PICOS).	5
**METHODS**			
Protocol and registration	5	Indicate if a review protocol exists, if and where it can be accessed (e.g., Web address), and, if available, provide registration information including registration number.	5
Eligibility criteria	6	Specify study characteristics (e.g., PICOS, length of follow-up) and report characteristics (e.g., years considered, language, publication status) used as criteria for eligibility, giving rationale.	6
Information sources	7	Describe all information sources (e.g., databases with dates of coverage, contact with study authors to identify additional studies) in the search and date last searched.	6
Search	8	Present full electronic search strategy for at least one database, including any limits used, such that it could be repeated.	Online supplement
Study selection	9	State the process for selecting studies (i.e., screening, eligibility, included in systematic review, and, if applicable, included in the meta-analysis).	6
Data collection process	10	Describe method of data extraction from reports (e.g., piloted forms, independently, in duplicate) and any processes for obtaining and confirming data from investigators.	6
Data items	11	List and define all variables for which data were sought (e.g., PICOS, funding sources) and any assumptions and simplifications made.	5,8
Risk of bias in individual studies	12	Describe methods used for assessing risk of bias of individual studies (including specification of whether this was done at the study or outcome level), and how this information is to be used in any data synthesis.	6
Summary measures	13	State the principal summary measures (e.g., risk ratio, difference in means).	7
Synthesis of results	14	Describe the methods of handling data and combining results of studies, if done, including measures of consistency (e.g., I^2) for each meta-analysis.	7, online supplement Box 2

Section/topic	#	Checklist item	Reported on page #
Risk of bias across studies	15	Specify any assessment of risk of bias that may affect the cumulative evidence (e.g., publication bias, selective reporting within studies).	7
Additional analyses	16	Describe methods of additional analyses (e.g., sensitivity or subgroup analyses, meta-regression), if done, indicating which were pre-specified.	7
**RESULTS**			
Study selection	17	Give numbers of studies screened, assessed for eligibility, and included in the review, with reasons for exclusions at each stage, ideally with a flow diagram.	8, Figure 1
Study characteristics	18	For each study, present characteristics for which data were extracted (e.g., study size, PICOS, follow-up period) and provide the citations.	8, online supplement-Table s1 and table S2
Risk of bias within studies	19	Present data on risk of bias of each study and, if available, any outcome level assessment (see item 12).	13, online supplement, Figure S1
Results of individual studies	20	For all outcomes considered (benefits or harms), present, for each study: (a) simple summary data for each intervention group (b) effect estimates and confidence intervals, ideally with a forest plot.	11, Fig 2 and 3
Synthesis of results	21	Present results of each meta-analysis done, including confidence intervals and measures of consistency.	11
Risk of bias across studies	22	Present results of any assessment of risk of bias across studies (see Item 15).	15
Additional analysis	23	Give results of additional analyses, if done (e.g., sensitivity or subgroup analyses, meta-regression [see Item 16]).	11,12,figures online supplement
**DISCUSSION**			
Summary of evidence	24	Summarize the main findings including the strength of evidence for each main outcome; consider their relevance to key groups (e.g., healthcare providers, users, and policy makers).	16-18
Limitations	25	Discuss limitations at study and outcome level (e.g., risk of bias), and at review-level (e.g., incomplete retrieval of identified research, reporting bias).	19-20
Conclusions	26	Provide a general interpretation of the results in the context of other evidence, and implications for future research.	20-21

**FUNDING**			
Funding	27	Describe sources of funding for the systematic review and other support (e.g., supply of data); role of funders for the systematic review.	8

From: Moher D, Liberati A, Tetzlaff J, Altman DG, The PRISMA Group (2009). Preferred Reporting Items for Systematic Reviews and Meta-Analyses: The PRISMA Statement. PLoS Med 6(7): e1000097. doi:10.1371/journal.pmed1000097

Figure S 1: Cochrane risk of bias assessment

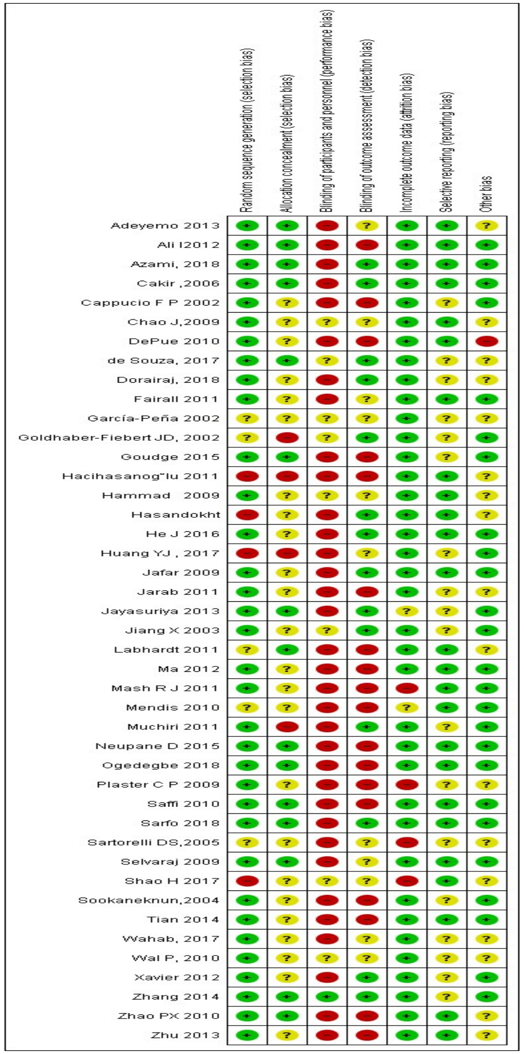

Figure S2: SBP Funnel Plot

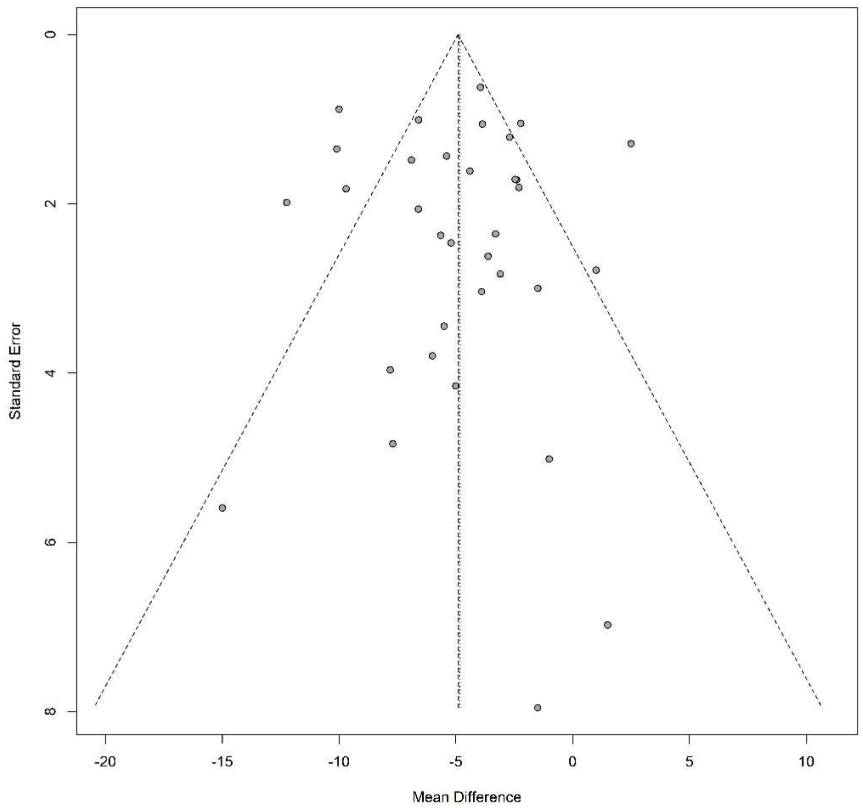

Figure S3: DBP Funnel Plot

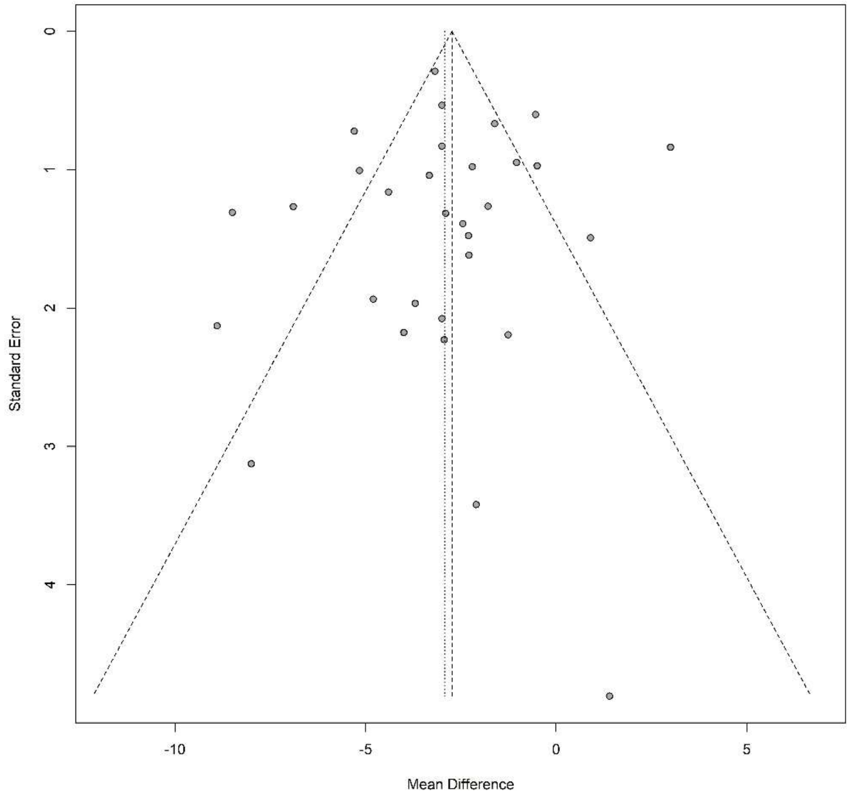

Figure S4: Sub group analysis by study population (SBP)

Study	Experimental Total	Experimental Mean	Experimental SD	Control Total	Control Mean	Control SD	MD	95%-CI	Weight
**Type II DM**									
Muchiri JW	41	-1.80	29.45	41	-3.30	33.59	1.50	[-12.17; 15.17]	0.7%
Mash R J	175	2.90	32.93	145	8.90	34.55	-6.00	[-13.45; 1.45]	1.8%
Ali MK	575	-18.30	25.43	571	-12.90	23.16	-5.40	[-8.22; -2.58]	3.9%
Jayasuriya R	28	-1.70	17.09	25	6.00	18.01	-7.70	[-17.18; 1.78]	1.3%
Hammad EA	110	-12.10	20.10	89	-6.90	14.60	-5.20	[-10.03; -0.37]	2.9%
Jarab AS	77	-5.80	11.64	79	1.10	5.89	-6.90	[-9.81; -3.99]	3.9%
Plaster CP	34	-7.00	3.50	29	3.00	3.50	-10.00	[-11.73; -8.27]	4.4%
Goldhaber-Fiebert JD	33	-5.00	23.00	28	-4.00	16.00	-1.00	[-10.83; 8.83]	1.2%
de Souza CF	62	-4.00	22.00	56	1.00	23.00	-5.00	[-13.14; 3.14]	1.6%
Azami G	71	-2.90	9.20	71	-0.60	12.15	-2.30	[-5.84; 1.24]	3.5%
Random effects model	1206			1134			-5.81	[-8.11; -3.50]	25.4%
Heterogeneity: $I^2 = 61\%$, $\tau^2 = 6.5205$, $p < 0.01$									
**CAD patients**									
Xavier D	375	0.30	22.60	375	2.70	24.39	-2.40	[-5.77; 0.97]	3.6%
Saffi MAL	38	-12.00	26.15	36	3.00	21.89	-15.00	[-25.97; -4.03]	1.1%
Jiang X	83	1.18	18.58	84	2.67	20.16	-1.49	[-7.37; 4.39]	2.4%
Zhang P	100	-14.10	11.30	99	-4.40	14.25	-9.70	[-13.28; -6.12]	3.5%
Random effects model	596			594			-6.16	[-11.42; -0.90]	10.6%
Heterogeneity: $I^2 = 77\%$, $\tau^2 = 20.3125$, $p < 0.01$									
**Hypertension**									
Zhu X	67	-14.72	20.02	67	-9.22	19.89	-5.50	[-12.26; 1.26]	2.1%
Jafar TH	269	-5.60	32.15	266	-6.60	32.29	1.00	[-4.46; 6.46]	2.6%
Mendis S I	256	-13.28	12.25	258	-9.42	11.77	-3.86	[-5.94; -1.78]	4.3%
Mendis S II	250	-11.01	15.37	255	-6.61	20.57	-4.40	[-7.56; -1.24]	3.7%
Tian M	1095	-11.80	27.90	991	-9.10	27.50	-2.70	[-5.08; -0.32]	4.1%
He J	709	-19.30	19.01	648	-12.70	18.18	-6.60	[-8.58; -4.62]	4.3%
Zhao PX	129	-8.50	16.45	129	-1.90	16.71	-6.60	[-10.65; -2.55]	3.3%
Cakir H	30	-8.90	5.20	30	1.20	5.30	-10.10	[-12.76; -7.44]	4.0%
Ma C	54	-11.36	20.83	52	-3.55	19.99	-7.81	[-15.58; -0.04]	1.7%
Neupane D [HTN]	118	-6.47	16.23	83	-2.85	19.63	-3.62	[-8.76; 1.52]	2.7%
Wal P	54	-30.70	10.42	48	-18.46	9.63	-12.24	[-16.13; -8.35]	3.3%
Sookaneknun P	118	-23.29	17.46	117	-17.64	18.91	-5.65	[-10.30; -1.00]	2.9%
Ogedegbe G	157	-19.80	21.16	166	-16.50	21.19	-3.30	[-7.92; 1.32]	3.0%
Sarfo FS	16	-9.00	24.30	16	-7.50	20.55	-1.50	[-17.09; 14.09]	0.6%
Prabhakaran D	331	-15.90	16.65	333	-18.40	16.65	2.50	[-0.03; 5.03]	4.1%
Random effects model	3653			3459			-4.81	[-6.96; -2.66]	46.7%
Heterogeneity: $I^2 = 81\%$, $\tau^2 = 12.7880$, $p < 0.01$									
**Community based**									
Chao J	957	-5.60	15.03	1005	-1.65	12.48	-3.95	[-5.18; -2.72]	4.6%
Neupane D [Normo]	197	4.39	9.11	141	6.61	9.82	-2.22	[-4.28; -0.16]	4.3%
Cappuccio FP	168	-1.30	26.20	169	1.80	25.75	-3.10	[-8.65; 2.45]	2.5%
Random effects model	1322			1315			-3.48	[-4.53; -2.43]	11.4%
Heterogeneity: $I^2 = 1\%$, $\tau^2 = 0.0127$, $p = 0.36$									
**High Risk/Obese adults**									
Neupane D [Pre HTN]	81	0.42	8.84	58	2.88	10.69	-2.46	[-5.82; 0.90]	3.6%
Sartorelli DS	40	-0.80	12.30	31	3.10	13.00	-3.90	[-9.86; 2.06]	2.4%
Random effects model	121			89			-2.81	[-5.73; 0.12]	6.0%
Heterogeneity: $I^2 = 0\%$, $\tau^2 = 0$, $p = 0.68$									
**Random effects model**	**6898**			**6591**			**-4.85**	**[-6.12; -3.57]**	**100.0%**
**Prediction interval**								**[-11.03; 1.33]**	
Heterogeneity: $I^2 = 76\%$, $\tau^2 = 8.7772$, $p < 0.01$									

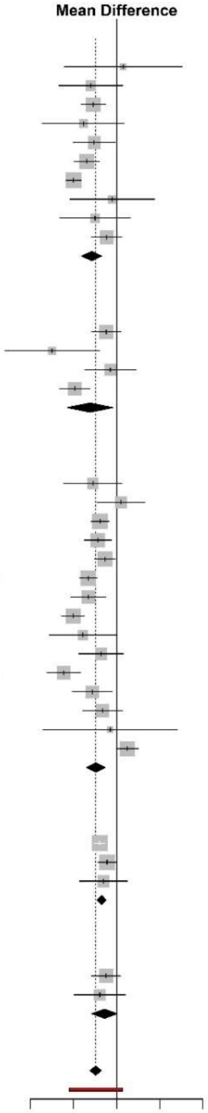

Figure S5: Sub group analysis by population (DBP)

Study	Experimental Total	Experimental Mean	Experimental SD	Control Total	Control Mean	Control SD	MD	95%-CI	Weight
**Type II DM**									
Muchiri JW	41	-4.50	15.49	41	-2.40	15.49	-2.10	[-8.81; 4.61]	1.2%
Mash R J	175	-0.90	16.66	145	2.80	18.18	-3.70	[-7.55; 0.15]	2.4%
Ali MK	575	-7.90	13.45	571	-4.90	14.62	-3.00	[-4.63; -1.37]	4.2%
Hammad EA	110	-7.20	12.60	89	-4.90	8.10	-2.30	[-5.19; 0.59]	3.1%
Jarab AS	77	-7.10	12.98	79	1.80	13.60	-8.90	[-13.07; -4.73]	2.2%
Plaster CP	34	-2.00	2.00	29	1.00	2.20	-3.00	[-4.05; -1.95]	4.7%
Goldhaber-Fiebert JD	33	-7.00	9.00	28	-3.00	8.00	-4.00	[-8.27; 0.27]	2.2%
de Souza CF	62	-1.00	11.00	56	2.00	11.50	-3.00	[-7.07; 1.07]	2.3%
Azami G	71	-2.40	5.20	71	-0.20	6.40	-2.20	[-4.12; -0.28]	4.0%
Random effects model	1178			1109			-3.10	[-3.93; -2.27]	26.3%
Heterogeneity: $I^2 = 11\%$, $\tau^2 = 0.1792$, $p = 0.35$									
**CAD patients**									
Xavier D	375	0.70	13.01	375	1.20	13.59	-0.50	[-2.40; 1.40]	4.0%
Saffi MAL	38	-5.00	13.44	36	3.00	13.44	-8.00	[-14.13; -1.87]	1.4%
Jiang X	83	0.09	13.67	84	1.35	14.66	-1.26	[-5.56; 3.04]	2.2%
Zhang P	100	-7.60	11.15	99	-0.70	5.99	-6.90	[-9.38; -4.42]	3.5%
Random effects model	596			594			-3.88	[-7.94; 0.18]	11.0%
Heterogeneity: $I^2 = 84\%$, $\tau^2 = 13.4816$, $p < 0.01$									
**Hypertension**									
Zhu X	67	-7.43	9.07	67	-5.14	9.65	-2.29	[-5.46; 0.88]	2.9%
Jafar TH	269	-4.80	17.22	266	-5.70	17.28	0.90	[-2.02; 3.82]	3.1%
Mendis S I	256	-6.07	7.54	258	-4.45	7.57	-1.62	[-2.92; -0.31]	4.5%
Mendis S II	250	-5.36	9.99	255	-2.03	13.20	-3.33	[-5.37; -1.29]	3.9%
He J	709	-12.20	13.58	648	-6.90	12.98	-5.30	[-6.71; -3.89]	4.4%
Zhao PX	129	-4.70	9.76	129	-1.80	11.31	-2.90	[-5.48; -0.32]	3.4%
Cakir H	30	-6.90	5.50	30	1.60	4.60	-8.50	[-11.07; -5.93]	3.4%
Ma C	54	-6.46	11.59	52	-3.52	11.36	-2.94	[-7.31; 1.43]	2.1%
Neupane D [HTN]	118	-2.90	7.84	83	-1.11	9.45	-1.79	[-4.27; 0.69]	3.5%
Wal P	54	-9.00	5.38	48	-3.84	4.79	-5.16	[-7.13; -3.19]	3.9%
Sookaneknun P	118	-14.18	11.20	117	-11.73	10.08	-2.45	[-5.17; 0.27]	3.3%
Sarfo FS	16	-3.80	15.34	16	-5.20	11.58	1.40	[-8.02; 10.82]	0.7%
Prabhakaran D	331	-6.50	11.10	333	-9.50	10.46	3.00	[1.36; 4.64]	4.2%
Random effects model	2401			2302			-2.58	[-4.37; -0.80]	43.2%
Heterogeneity: $I^2 = 87\%$, $\tau^2 = 8.6598$, $p < 0.01$									
**Community based**									
Chao J	957	-3.76	6.80	1005	-0.58	5.89	-3.18	[-3.74; -2.62]	4.9%
Neupane D [Normo]	197	4.58	5.36	141	5.12	5.52	-0.54	[-1.72; 0.64]	4.6%
Cappuccio FP	168	-0.90	10.59	169	3.50	10.73	-4.40	[-6.68; -2.12]	3.7%
Random effects model	1322			1315			-2.58	[-4.63; -0.53]	13.1%
Heterogeneity: $I^2 = 89\%$, $\tau^2 = 2.7643$, $p < 0.01$									
**High Risk/Obese adults**									
Neupane D [Pre HTN]	81	1.48	5.18	58	2.52	5.74	-1.04	[-2.90; 0.82]	4.0%
Sartorelli DS	40	-1.30	8.90	31	3.50	7.40	-4.80	[-8.59; -1.01]	2.5%
Random effects model	121			89			-2.54	[-6.15; 1.07]	6.5%
Heterogeneity: $I^2 = 67\%$, $\tau^2 = 4.7459$, $p = 0.08$									
**Random effects model**	**5618**			**5409**			**-2.92**	**[-3.75; -2.09]**	**100.0%**
**Prediction interval**								**[-6.90; 1.06]**	
Heterogeneity: $I^2 = 80\%$, $\tau^2 = 3.6077$, $p < 0.01$									

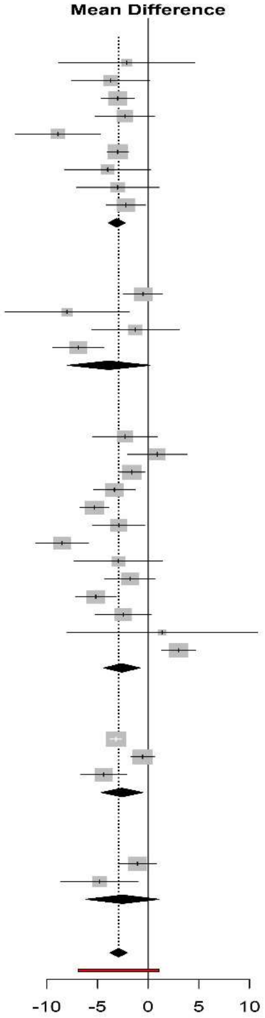

Figure S6: Sub group analysis by follow-up duration (SBP)

Study	Experimental Total	Experimental Mean	Experimental SD	Control Total	Control Mean	Control SD	MD	95%-CI	Weight
**0-6 months**									
Jiang X	83	1.18	18.58	84	2.67	20.16	-1.49	[-7.37; 4.39]	2.4%
Zhu X	67	-14.72	20.02	67	-9.22	19.89	-5.50	[-12.26; 1.26]	2.1%
Jayasuriya R	28	-1.70	17.09	25	6.00	18.01	-7.70	[-17.18; 1.78]	1.3%
Hammad EA	110	-12.10	20.10	89	-6.90	14.60	-5.20	[-10.03; -0.37]	2.9%
Jarab AS	77	-5.80	11.64	79	1.10	5.89	-6.90	[-9.81; -3.99]	3.9%
Zhao PX	129	-8.50	16.45	129	-1.90	16.71	-6.60	[-10.65; -2.55]	3.3%
Plaster CP	34	-7.00	3.50	29	3.00	3.50	-10.00	[-11.73; -8.27]	4.4%
Cakir H	30	-8.90	5.20	30	1.20	5.30	-10.10	[-12.76; -7.44]	4.0%
Ma C	54	-11.36	20.83	52	-3.55	19.99	-7.81	[-15.58; -0.04]	1.7%
Cappuccio FP	168	-1.30	26.20	169	1.80	25.75	-3.10	[-8.65; 2.45]	2.5%
Goldhaber-Fiebert JD	33	-5.00	23.00	28	-4.00	16.00	-1.00	[-10.83; 8.83]	1.2%
Wal P	54	-30.70	10.42	48	-18.46	9.63	-12.24	[-16.13; -8.35]	3.3%
Sookaneknun P	118	-23.29	17.46	117	-17.64	18.91	-5.65	[-10.30; -1.00]	2.9%
de Souza CF	62	-4.00	22.00	56	1.00	23.00	-5.00	[-13.14; 3.14]	1.6%
Azami G	71	-2.90	9.20	71	-0.60	12.15	-2.30	[-5.84; 1.24]	3.5%
Random effects model	1118			1073			-6.64	[-8.46; -4.81]	41.1%
Heterogeneity: $I^2 = 61\%$, $\tau^2 = 6.5988$, $p < 0.01$									
**7-12 months**									
Muchiri JW	41	-1.80	29.45	41	-3.30	33.59	1.50	[-12.17; 15.17]	0.7%
Mash R J	175	2.90	32.93	145	8.90	34.55	-6.00	[-13.45; 1.45]	1.8%
Xavier D	375	0.30	22.60	375	2.70	24.39	-2.40	[-5.77; 0.97]	3.6%
Saffi MAL	38	-12.00	26.15	36	3.00	21.89	-15.00	[-25.97; -4.03]	1.1%
Mendis S I	256	-13.28	12.25	258	-9.42	11.77	-3.86	[-5.94; -1.78]	4.3%
Mendis S II	250	-11.01	15.37	255	-6.61	20.57	-4.40	[-7.56; -1.24]	3.7%
Tian M	1095	-11.80	27.90	991	-9.10	27.50	-2.70	[-5.08; -0.32]	4.1%
Neupane D [Normo]	197	4.39	9.11	141	6.61	9.82	-2.22	[-4.28; -0.16]	4.3%
Neupane D [Pre HTN]	81	0.42	8.84	58	2.88	10.69	-2.46	[-5.82; 0.90]	3.6%
Neupane D [HTN]	118	-6.47	16.23	83	-2.85	19.63	-3.62	[-8.76; 1.52]	2.7%
Zhang P	100	-14.10	11.30	99	-4.40	14.25	-9.70	[-13.28; -6.12]	3.5%
Sartorelli DS	40	-0.80	12.30	31	3.10	13.00	-3.90	[-9.86; 2.06]	2.4%
Ogedegbe G	157	-19.80	21.16	166	-16.50	21.19	-3.30	[-7.92; 1.32]	3.0%
Sarfo FS	16	-9.00	24.30	16	-7.50	20.55	-1.50	[-17.09; 14.09]	0.6%
Prabhakaran D	331	-15.90	16.65	333	-18.40	16.65	2.50	[-0.03; 5.03]	4.1%
Random effects model	3270			3028			-3.37	[-5.05; -1.69]	43.5%
Heterogeneity: $I^2 = 65\%$, $\tau^2 = 5.8029$, $p < 0.01$									
**13-24 months**									
Ali MK	575	-18.30	25.43	571	-12.90	23.16	-5.40	[-8.22; -2.58]	3.9%
Jafar TH	269	-5.60	32.15	266	-6.60	32.29	1.00	[-4.46; 6.46]	2.6%
He J	709	-19.30	19.01	648	-12.70	18.18	-6.60	[-8.58; -4.62]	4.3%
Chao J	957	-5.60	15.03	1005	-1.65	12.48	-3.95	[-5.18; -2.72]	4.6%
Random effects model	2510			2490			-4.55	[-6.64; -2.46]	15.4%
Heterogeneity: $I^2 = 68\%$, $\tau^2 = 2.7819$, $p = 0.02$									
**Random effects model**	**6898**			**6591**			**-4.85**	**[-6.12; -3.57]**	**100.0%**
**Prediction interval**								**[-11.03; 1.33]**	
Heterogeneity: $I^2 = 76\%$, $\tau^2 = 8.7772$, $p < 0.01$									

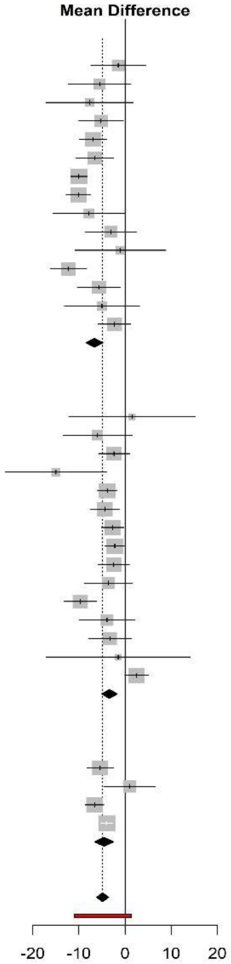

Figure S7: Sub group analyses by follow-up duration (DBP)

Study	Experimental Total	Experimental Mean	Experimental SD	Control Total	Control Mean	Control SD	MD	95%-CI	Weight
**0-6 months**									
Jiang X	83	0.09	13.67	84	1.35	14.66	-1.26	[-5.56; 3.04]	2.2%
Zhu X	67	-7.43	9.07	67	-5.14	9.65	-2.29	[-5.46; 0.88]	2.9%
Hammad EA	110	-7.20	12.60	89	-4.90	8.10	-2.30	[-5.19; 0.59]	3.1%
Jarab AS	77	-7.10	12.98	79	1.80	13.60	-8.90	[-13.07; -4.73]	2.2%
Zhao PX	129	-4.70	9.76	129	-1.80	11.31	-2.90	[-5.48; -0.32]	3.4%
Plaster CP	34	-2.00	2.00	29	1.00	2.20	-3.00	[-4.05; -1.95]	4.7%
Cakir H	30	-6.90	5.50	30	1.60	4.60	-8.50	[-11.07; -5.93]	3.4%
Ma C	54	-6.46	11.59	52	-3.52	11.36	-2.94	[-7.31; 1.43]	2.1%
Cappuccio FP	168	-0.90	10.59	169	3.50	10.73	-4.40	[-6.68; -2.12]	3.7%
Goldhaber-Fiebert JD	33	-7.00	9.00	28	-3.00	8.00	-4.00	[-8.27; 0.27]	2.2%
Wal P	54	-9.00	5.38	48	-3.84	4.79	-5.16	[-7.13; -3.19]	3.9%
Sookaneknun P	118	-14.18	11.20	117	-11.73	10.08	-2.45	[-5.17; 0.27]	3.3%
de Souza CF	62	-1.00	11.00	56	2.00	11.50	-3.00	[-7.07; 1.07]	2.3%
Azami G	71	-2.40	5.20	71	-0.20	6.40	-2.20	[-4.12; -0.28]	4.0%
Random effects model	1090			1048			-3.78	[-4.85; -2.70]	43.2%
Heterogeneity: $I^2 = 57\%$, $\tau^2 = 2.0876$, $p < 0.01$									
**7-12 months**									
Muchiri JW	41	-4.50	15.49	41	-2.40	15.49	-2.10	[-8.81; 4.61]	1.2%
Mash R J	175	-0.90	16.66	145	2.80	18.18	-3.70	[-7.55; 0.15]	2.4%
Xavier D	375	0.70	13.01	375	1.20	13.59	-0.50	[-2.40; 1.40]	4.0%
Saffi MAL	38	-5.00	13.44	36	3.00	13.44	-8.00	[-14.13; -1.87]	1.4%
Mendis S I	256	-6.07	7.54	258	-4.45	7.57	-1.62	[-2.92; -0.31]	4.5%
Mendis S II	250	-5.36	9.99	255	-2.03	13.20	-3.33	[-5.37; -1.29]	3.9%
Neupane D [Normo]	197	4.58	5.36	141	5.12	5.52	-0.54	[-1.72; 0.64]	4.6%
Neupane D [Pre HTN]	81	1.48	5.18	58	2.52	5.74	-1.04	[-2.90; 0.82]	4.0%
Neupane D [HTN]	118	-2.90	7.84	83	-1.11	9.45	-1.79	[-4.27; 0.69]	3.5%
Zhang P	100	-7.60	11.15	99	-0.70	5.99	-6.90	[-9.38; -4.42]	3.5%
Sartorelli DS	40	-1.30	8.90	31	3.50	7.40	-4.80	[-8.59; -1.01]	2.5%
Sarfo FS	16	-3.80	15.34	16	-5.20	11.58	1.40	[-8.02; 10.82]	0.7%
Prabhakaran D	331	-6.50	11.10	333	-9.50	10.46	3.00	[1.36; 4.64]	4.2%
Random effects model	2018			1871			-2.06	[-3.55; -0.57]	40.1%
Heterogeneity: $I^2 = 81\%$, $\tau^2 = 5.0584$, $p < 0.01$									
**13-24 months**									
Ali MK	575	-7.90	13.45	571	-4.90	14.62	-3.00	[-4.63; -1.37]	4.2%
Jafar TH	269	-4.80	17.22	266	-5.70	17.28	0.90	[-2.02; 3.82]	3.1%
He J	709	-12.20	13.58	648	-6.90	12.98	-5.30	[-6.71; -3.89]	4.4%
Chao J	957	-3.76	6.80	1005	-0.58	5.89	-3.18	[-3.74; -2.62]	4.9%
Random effects model	2510			2490			-3.05	[-4.67; -1.43]	16.6%
Heterogeneity: $I^2 = 81\%$, $\tau^2 = 2.0353$, $p < 0.01$									
**Random effects model**	**5618**			**5409**			**-2.92**	**[-3.75; -2.09]**	**100.0%**
**Prediction interval**								**[-6.90; 1.06]**	
Heterogeneity: $I^2 = 80\%$, $\tau^2 = 3.6077$, $p < 0.01$									

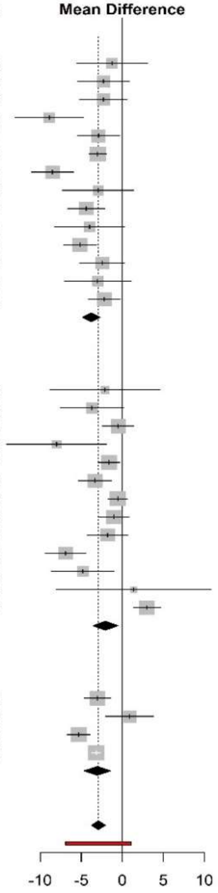

Figure S8: Sub group analyses by sample size (SBP)

Study	Experimental Total	Experimental Mean	Experimental SD	Control Total	Control Mean	Control SD	MD	95%-CI	Weight
**0-500 study participants**									
Muchiri JW	41	-1.80	29.45	41	-3.30	33.59	1.50	[-12.17; 15.17]	0.7%
Mash R J	175	2.90	32.93	145	8.90	34.55	-6.00	[-13.45; 1.45]	1.8%
Saffi MAL	38	-12.00	26.15	36	3.00	21.89	-15.00	[-25.97; -4.03]	1.1%
Jiang X	83	1.18	18.58	84	2.67	20.16	-1.49	[-7.37; 4.39]	2.4%
Zhu X	67	-14.72	20.02	67	-9.22	19.89	-5.50	[-12.26; 1.26]	2.1%
Jayasuriya R	28	-1.70	17.09	25	6.00	18.01	-7.70	[-17.18; 1.78]	1.3%
Hammad EA	110	-12.10	20.10	89	-6.90	14.60	-5.20	[-10.03; -0.37]	2.9%
Jarab AS	77	-5.80	11.64	79	1.10	5.89	-6.90	[-9.81; -3.99]	3.9%
Zhao PX	129	-8.50	16.45	129	-1.90	16.71	-6.60	[-10.65; -2.55]	3.3%
Plaster CP	34	-7.00	3.50	29	3.00	3.50	-10.00	[-11.73; -8.27]	4.4%
Cakir H	30	-8.90	5.20	30	1.20	5.30	-10.10	[-12.76; -7.44]	4.0%
Ma C	54	-11.36	20.83	52	-3.55	19.99	-7.81	[-15.58; -0.04]	1.7%
Neupane D [Normo]	197	4.39	9.11	141	6.61	9.82	-2.22	[-4.28; -0.16]	4.3%
Neupane D [Pre HTN]	81	0.42	8.84	58	2.88	10.69	-2.46	[-5.82; 0.90]	3.6%
Neupane D [HTN]	118	-6.47	16.23	83	-2.85	19.63	-3.62	[-8.76; 1.52]	2.7%
Cappuccio FP	168	-1.30	26.20	169	1.80	25.75	-3.10	[-8.65; 2.45]	2.5%
Goldhaber-Fiebert JD	33	-5.00	23.00	28	-4.00	16.00	-1.00	[-10.83; 8.83]	1.2%
Wal P	54	-30.70	10.42	48	-18.46	9.63	-12.24	[-16.13; -8.35]	3.3%
Sookaneknun P	118	-23.29	17.46	117	-17.64	18.91	-5.65	[-10.30; -1.00]	2.9%
Zhang P	100	-14.10	11.30	99	-4.40	14.25	-9.70	[-13.28; -6.12]	3.5%
Sartorelli DS	40	-0.80	12.30	31	3.10	13.00	-3.90	[-9.86; 2.06]	2.4%
Ogedegbe G	157	-19.80	21.16	166	-16.50	21.19	-3.30	[-7.92; 1.32]	3.0%
Sarfo FS	16	-9.00	24.30	16	-7.50	20.55	-1.50	[-17.09; 14.09]	0.6%
de Souza CF	62	-4.00	22.00	56	1.00	23.00	-5.00	[-13.14; 3.14]	1.6%
Azami G	71	-2.90	9.20	71	-0.60	12.15	-2.30	[-5.84; 1.24]	3.5%
**Random effects model**	**2081**			**1889**			**-5.81**	**[-7.43; -4.18]**	**64.7%**
Heterogeneity: $I^2 = 68\%$, $\tau^2 = 9.4665$, $p < 0.01$									
**>500 Study participants**									
Xavier D	375	0.30	22.60	375	2.70	24.39	-2.40	[-5.77; 0.97]	3.6%
Ali MK	575	-18.30	25.43	571	-12.90	23.16	-5.40	[-8.22; -2.58]	3.9%
Jafar TH	269	-5.60	32.15	266	-6.60	32.29	1.00	[-4.46; 6.46]	2.6%
Mendis S I	256	-13.28	12.25	258	-9.42	11.77	-3.86	[-5.94; -1.78]	4.3%
Mendis S II	250	-11.01	15.37	255	-6.61	20.57	-4.40	[-7.56; -1.24]	3.7%
Tian M	1095	-11.80	27.90	991	-9.10	27.50	-2.70	[-5.08; -0.32]	4.1%
He J	709	-19.30	19.01	648	-12.70	18.18	-6.60	[-8.58; -4.62]	4.3%
Chao J	957	-5.60	15.03	1005	-1.65	12.48	-3.95	[-5.18; -2.72]	4.6%
Prabhakaran D	331	-15.90	16.65	333	-18.40	16.65	2.50	[-0.03; 5.03]	4.1%
**Random effects model**	**4817**			**4702**			**-3.13**	**[-4.87; -1.40]**	**35.3%**
Heterogeneity: $I^2 = 78\%$, $\tau^2 = 5.1207$, $p < 0.01$									
**Random effects model**	**6898**			**6591**			**-4.85**	**[-6.12; -3.57]**	**100.0%**
**Prediction interval**								**[-11.03; 1.33]**	
Heterogeneity: $I^2 = 76\%$, $\tau^2 = 8.7772$, $p < 0.01$									

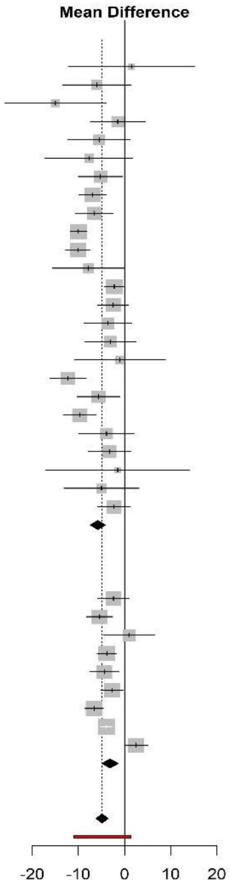

Figure S9: Sub group analysis- SBP (Sample Size)

Study	Experimental Total	Experimental Mean	Experimental SD	Control Total	Control Mean	Control SD	MD	95%-CI	Weight
**0-500 Study participants**									
Muchiri JW	41	-4.50	15.49	41	-2.40	15.49	-2.10	[-8.81; 4.61]	1.2%
Mash R J	175	-0.90	16.66	145	2.80	18.18	-3.70	[-7.55; 0.15]	2.4%
Saffi MAL	38	-5.00	13.44	36	3.00	13.44	-8.00	[-14.13; -1.87]	1.4%
Jiang X	83	0.09	13.67	84	1.35	14.66	-1.26	[-5.56; 3.04]	2.2%
Zhu X	67	-7.43	9.07	67	-5.14	9.65	-2.29	[-5.46; 0.88]	2.9%
Hammad EA	110	-7.20	12.60	89	-4.90	8.10	-2.30	[-5.19; 0.59]	3.1%
Jarab AS	77	-7.10	12.98	79	1.80	13.60	-8.90	[-13.07; -4.73]	2.2%
Zhao PX	129	-4.70	9.76	129	-1.80	11.31	-2.90	[-5.48; -0.32]	3.4%
Plaster CP	34	-2.00	2.00	29	1.00	2.20	-3.00	[-4.05; -1.95]	4.7%
Cakir H	30	-6.90	5.50	30	1.60	4.60	-8.50	[-11.07; -5.93]	3.4%
Ma C	54	-6.46	11.59	52	-3.52	11.36	-2.94	[-7.31; 1.43]	2.1%
Neupane D [Normo]	197	4.58	5.36	141	5.12	5.52	-0.54	[-1.72; 0.64]	4.6%
Neupane D [Pre HTN]	81	1.48	5.18	58	2.52	5.74	-1.04	[-2.90; 0.82]	4.0%
Neupane D [HTN]	118	-2.90	7.84	83	-1.11	9.45	-1.79	[-4.27; 0.69]	3.5%
Cappuccio FP	168	-0.90	10.59	169	3.50	10.73	-4.40	[-6.68; -2.12]	3.7%
Goldhaber-Fiebert JD	33	-7.00	9.00	28	-3.00	8.00	-4.00	[-8.27; 0.27]	2.2%
Wal P	54	-9.00	5.38	48	-3.84	4.79	-5.16	[-7.13; -3.19]	3.9%
Sookaneknun P	118	-14.18	11.20	117	-11.73	10.08	-2.45	[-5.17; 0.27]	3.3%
Zhang P	100	-7.60	11.15	99	-0.70	5.99	-6.90	[-9.38; -4.42]	3.5%
Sartorelli DS	40	-1.30	8.90	31	3.50	7.40	-4.80	[-8.59; -1.01]	2.5%
Sarfo FS	16	-3.80	15.34	16	-5.20	11.58	1.40	[-8.02; 10.82]	0.7%
de Souza CF	62	-1.00	11.00	56	2.00	11.50	-3.00	[-7.07; 1.07]	2.3%
Azami G	71	-2.40	5.20	71	-0.20	6.40	-2.20	[-4.12; -0.28]	4.0%
**Random effects model**	1896			1698			-3.49	[-4.49; -2.49]	66.9%
Heterogeneity: $I^2 = 68\%$, $\tau^2 = 3.3569$, $p < 0.01$									
**>500 Study participants**									
Xavier D	375	0.70	13.01	375	1.20	13.59	-0.50	[-2.40; 1.40]	4.0%
Ali MK	575	-7.90	13.45	571	-4.90	14.62	-3.00	[-4.63; -1.37]	4.2%
Jafar TH	269	-4.80	17.22	266	-5.70	17.28	0.90	[-2.02; 3.82]	3.1%
Mendis S I	256	-6.07	7.54	258	-4.45	7.57	-1.62	[-2.92; -0.31]	4.5%
Mendis S II	250	-5.36	9.99	255	-2.03	13.20	-3.33	[-5.37; -1.29]	3.9%
He J	709	-12.20	13.58	648	-6.90	12.98	-5.30	[-6.71; -3.89]	4.4%
Chao J	957	-3.76	6.80	1005	-0.58	5.89	-3.18	[-3.74; -2.62]	4.9%
Prabhakaran D	331	-6.50	11.10	333	-9.50	10.46	3.00	[1.36; 4.64]	4.2%
**Random effects model**	3722			3711			-1.73	[-3.38; -0.08]	33.1%
Heterogeneity: $I^2 = 91\%$, $\tau^2 = 4.8872$, $p < 0.01$									
**Random effects model**	**5618**			**5409**			**-2.92**	**[-3.75; -2.09]**	**100.0%**
**Prediction interval**								**[-6.90; 1.06]**	
Heterogeneity: $I^2 = 80\%$, $\tau^2 = 3.6077$, $p < 0.01$									

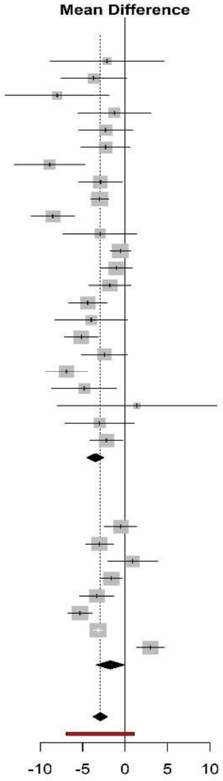

Figure S10: Sub group analysis by level of health system capacity (SBP)

Study	Experimental Total	Experimental Mean	Experimental SD	Control Total	Control Mean	Control SD	MD	95%-CI	Weight
**0-5 Physician/10,000**									
Neupane D [Normo]	197	4.39	9.11	141	6.61	9.82	-2.22	[-4.28; -0.16]	4.3%
Neupane D [Pre HTN]	81	0.42	8.84	58	2.88	10.69	-2.46	[-5.82; 0.90]	3.6%
Neupane D [HTN]	118	-6.47	16.23	83	-2.85	19.63	-3.62	[-8.76; 1.52]	2.7%
Cappuccio FP	168	-1.30	26.20	169	1.80	25.75	-3.10	[-8.65; 2.45]	2.5%
Sookaneknun P	118	-23.29	17.46	117	-17.64	18.91	-5.65	[-10.30; -1.00]	2.9%
Ogedegbe G	157	-19.80	21.16	166	-16.50	21.19	-3.30	[-7.92; 1.32]	3.0%
Sarfo FS	16	-9.00	24.30	16	-7.50	20.55	-1.50	[-17.09; 14.09]	0.6%
Azami G	71	-2.90	9.20	71	-0.60	12.15	-2.30	[-5.84; 1.24]	3.5%
**Random effects model**	**926**			**821**			**-2.77**	**[-4.09; -1.45]**	**23.2%**
Heterogeneity: $I^2 = 0\%$, $\tau^2 = 0$, $p = 0.96$									
**6-10 Physician/10,000**									
Muchiri JW	41	-1.80	29.45	41	-3.30	33.59	1.50	[-12.17; 15.17]	0.7%
Mash R J	175	2.90	32.93	145	8.90	34.55	-6.00	[-13.45; 1.45]	1.8%
Xavier D	375	0.30	22.60	375	2.70	24.39	-2.40	[-5.77; 0.97]	3.6%
Ali MK	575	-18.30	25.43	571	-12.90	23.16	-5.40	[-8.22; -2.58]	3.9%
Jafar TH	269	-5.60	32.15	266	-6.60	32.29	1.00	[-4.46; 6.46]	2.6%
Jayasuriya R	28	-1.70	17.09	25	6.00	18.01	-7.70	[-17.18; 1.78]	1.3%
Wal P	54	-30.70	10.42	48	-18.46	9.63	-12.24	[-16.13; -8.35]	3.3%
Prabhakaran D	331	-15.90	16.65	333	-18.40	16.65	2.50	[-0.03; 5.03]	4.1%
**Random effects model**	**1848**			**1804**			**-3.73**	**[-7.83; 0.38]**	**21.4%**
Heterogeneity: $I^2 = 85\%$, $\tau^2 = 26.0119$, $p < 0.01$									
**11-15 Physician/10,000**									
Jiang X	83	1.18	18.58	84	2.67	20.16	-1.49	[-7.37; 4.39]	2.4%
Zhu X	67	-14.72	20.02	67	-9.22	19.89	-5.50	[-12.26; 1.26]	2.1%
Mendis S I	256	-13.28	12.25	258	-9.42	11.77	-3.86	[-5.94; -1.78]	4.3%
Mendis S II	250	-11.01	15.37	255	-6.61	20.57	-4.40	[-7.56; -1.24]	3.7%
Tian M	1095	-11.80	27.90	991	-9.10	27.50	-2.70	[-5.08; -0.32]	4.1%
Zhao PX	129	-8.50	16.45	129	-1.90	16.71	-6.60	[-10.65; -2.55]	3.3%
Chao J	957	-5.60	15.03	1005	-1.65	12.48	-3.95	[-5.18; -2.72]	4.6%
Ma C	54	-11.36	20.83	52	-3.55	19.99	-7.81	[-15.58; -0.04]	1.7%
Goldhaber-Fiebert JD	33	-5.00	23.00	28	-4.00	16.00	-1.00	[-10.83; 8.83]	1.2%
Zhang P	100	-14.10	11.30	99	-4.40	14.25	-9.70	[-13.28; -6.12]	3.5%
**Random effects model**	**3024**			**2968**			**-4.55**	**[-5.87; -3.23]**	**30.9%**
Heterogeneity: $I^2 = 38\%$, $\tau^2 = 1.4188$, $p = 0.11$									
**Above 15Physician/10,000**									
Saffi MAL	38	-12.00	26.15	36	3.00	21.89	-15.00	[-25.97; -4.03]	1.1%
Hammad EA	110	-12.10	20.10	89	-6.90	14.60	-5.20	[-10.03; -0.37]	2.9%
He J	709	-19.30	19.01	648	-12.70	18.18	-6.60	[-8.58; -4.62]	4.3%
Jarab AS	77	-5.80	11.64	79	1.10	5.89	-6.90	[-9.81; -3.99]	3.9%
Plaster CP	34	-7.00	3.50	29	3.00	3.50	-10.00	[-11.73; -8.27]	4.4%
Cakir H	30	-8.90	5.20	30	1.20	5.30	-10.10	[-12.76; -7.44]	4.0%
Sartorelli DS	40	-0.80	12.30	31	3.10	13.00	-3.90	[-9.86; 2.06]	2.4%
de Souza CF	62	-4.00	22.00	56	1.00	23.00	-5.00	[-13.14; 3.14]	1.6%
**Random effects model**	**1100**			**998**			**-7.85**	**[-9.63; -6.07]**	**24.5%**
Heterogeneity: $I^2 = 53\%$, $\tau^2 = 2.8780$, $p = 0.04$									
**Random effects model**	**6898**			**6591**			**-4.85**	**[-6.12; -3.57]**	**100.0%**
**Prediction interval**								**[-11.03; 1.33]**	
Heterogeneity: $I^2 = 76\%$, $\tau^2 = 8.7772$, $p < 0.01$									

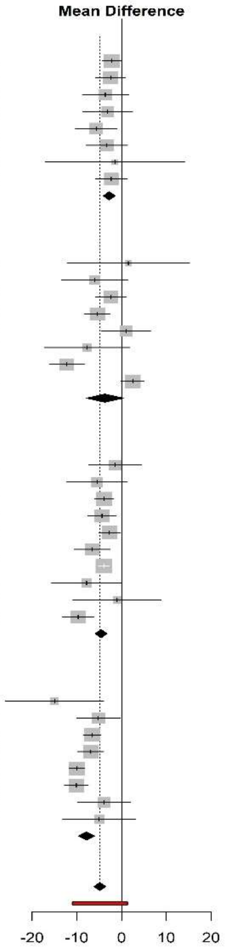

Fig S11: Sub group analyses by level of health system capacity (DBP)

Study	Experimental Total	Experimental Mean	Experimental SD	Control Total	Control Mean	Control SD	MD	95%-CI	Weight
**0-5 Physician/10,000**									
Neupane D [Normo]	197	4.58	5.36	141	5.12	5.52	-0.54	[-1.72; 0.64]	4.6%
Neupane D [Pre HTN]	81	1.48	5.18	58	2.52	5.74	-1.04	[-2.90; 0.82]	4.0%
Neupane D [HTN]	118	-2.90	7.84	83	-1.11	9.45	-1.79	[-4.27; 0.69]	3.5%
Cappuccio FP	168	-0.90	10.59	169	3.50	10.73	-4.40	[-6.68; -2.12]	3.7%
Sookaneknun P	118	-14.18	11.20	117	-11.73	10.08	-2.45	[-5.17; 0.27]	3.3%
Sarfo FS	16	-3.80	15.34	16	-5.20	11.58	1.40	[-8.02; 10.82]	0.7%
Azami G	71	-2.40	5.20	71	-0.20	6.40	-2.20	[-4.12; -0.28]	4.0%
**Random effects model**	**769**			**655**			**-1.81**	**[-2.90; -0.72]**	**23.6%**
Heterogeneity: $I^2 = 42\%$, $\tau^2 = 0.8441$, $p = 0.11$									
**6-10 Physician/10,000**									
Muchiri JW	41	-4.50	15.49	41	-2.40	15.49	-2.10	[-8.81; 4.61]	1.2%
Mash R J	175	-0.90	16.66	145	2.80	18.18	-3.70	[-7.55; 0.15]	2.4%
Xavier D	375	0.70	13.01	375	1.20	13.59	-0.50	[-2.40; 1.40]	4.0%
Ali MK	575	-7.90	13.45	571	-4.90	14.62	-3.00	[-4.63; -1.37]	4.2%
Jafar TH	269	-4.80	17.22	266	-5.70	17.28	0.90	[-2.02; 3.82]	3.1%
Wal P	54	-9.00	5.38	48	-3.84	4.79	-5.16	[-7.13; -3.19]	3.9%
Prabhakaran D	331	-6.50	11.10	333	-9.50	10.46	3.00	[1.36; 4.64]	4.2%
**Random effects model**	**1820**			**1779**			**-1.41**	**[-3.95; 1.13]**	**23.0%**
Heterogeneity: $I^2 = 88\%$, $\tau^2 = 9.5004$, $p < 0.01$									
**11-15 Physician/10,000**									
Jiang X	83	0.09	13.67	84	1.35	14.66	-1.26	[-5.56; 3.04]	2.2%
Zhu X	67	-7.43	9.07	67	-5.14	9.65	-2.29	[-5.46; 0.88]	2.9%
Mendis S I	256	-6.07	7.54	258	-4.45	7.57	-1.62	[-2.92; -0.31]	4.5%
Mendis S II	250	-5.36	9.99	255	-2.03	13.20	-3.33	[-5.37; -1.29]	3.9%
Zhao PX	129	-4.70	9.76	129	-1.80	11.31	-2.90	[-5.48; -0.32]	3.4%
Chao J	957	-3.76	6.80	1005	-0.58	5.89	-3.18	[-3.74; -2.62]	4.9%
Ma C	54	-6.46	11.59	52	-3.52	11.36	-2.94	[-7.31; 1.43]	2.1%
Goldhaber-Fiebert JD	33	-7.00	9.00	28	-3.00	8.00	-4.00	[-8.27; 0.27]	2.2%
Zhang P	100	-7.60	11.15	99	-0.70	5.99	-6.90	[-9.38; -4.42]	3.5%
**Random effects model**	**1929**			**1977**			**-3.12**	**[-4.10; -2.15]**	**29.4%**
Heterogeneity: $I^2 = 47\%$, $\tau^2 = 0.8295$, $p = 0.06$									
**Above 15Physician/10,000**									
Saffi MAL	38	-5.00	13.44	36	3.00	13.44	-8.00	[-14.13; -1.87]	1.4%
Hammad EA	110	-7.20	12.60	89	-4.90	8.10	-2.30	[-5.19; 0.59]	3.1%
He J	709	-12.20	13.58	648	-6.90	12.98	-5.30	[-6.71; -3.89]	4.4%
Jarab AS	77	-7.10	12.98	79	1.80	13.60	-8.90	[-13.07; -4.73]	2.2%
Plaster CP	34	-2.00	2.00	29	1.00	2.20	-3.00	[-4.05; -1.95]	4.7%
Cakir H	30	-6.90	5.50	30	1.60	4.60	-8.50	[-11.07; -5.93]	3.4%
Sartorelli DS	40	-1.30	8.90	31	3.50	7.40	-4.80	[-8.59; -1.01]	2.5%
de Souza CF	62	-1.00	11.00	56	2.00	11.50	-3.00	[-7.07; 1.07]	2.3%
**Random effects model**	**1100**			**998**			**-5.15**	**[-6.90; -3.39]**	**23.9%**
Heterogeneity: $I^2 = 74\%$, $\tau^2 = 3.9020$, $p < 0.01$									
**Random effects model**	**5618**			**5409**			**-2.92**	**[-3.75; -2.09]**	**100.0%**
**Prediction interval**								**[-6.90; 1.06]**	
Heterogeneity: $I^2 = 80\%$, $\tau^2 = 3.6077$, $p < 0.01$									

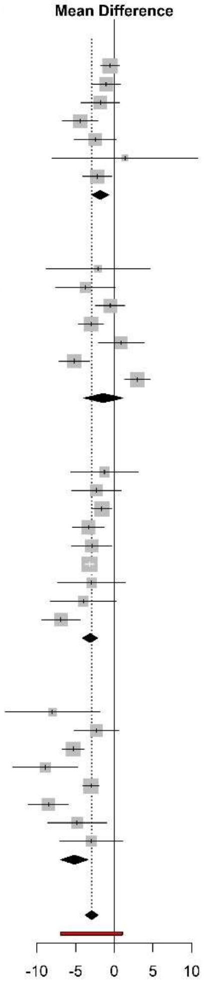

Box 1

Run on 18.July. 2017

CVD: “Hyperlipidemias”[MeSH] OR “hyperlipidemias”[All Fields] OR “hyperlipidemia”[All Fields] OR “hyperlipidaemia”[All Fields] OR “hyperlipidaemias”[All Fields] OR “hyperlipemia”[All Fields] OR “hyperlipemias”[All Fields] OR “hyperlipaemia”[All Fields] OR “hyperlipaemias”[All Fields] OR “lipidemia”[All Fields] OR “lipidaemia”[All Fields] OR “high cholesterol”[All Fields] OR “hypercholesterolemia”[All Fields] OR “hypercholesterolemias”[All Fields] OR “hypercholesteremia”[All Fields] OR “hypercholesteremias”[All Fields] OR “hypercholesterolaemia”[All Fields] OR “hypercholesterolaemias”[All Fields] OR “hypercholesteraemia”[All Fields] OR “Diabetes”[All Fields] OR “diabetic”[All fields] OR "Diabetes Mellitus"[Mesh] OR “proteinuria”[Mesh] OR “proteinuria”[All Fields] OR “Albuminuria”[All Fields] OR “Hemoglobinuria”[All Fields] OR "Kidney Failure, Chronic"[Mesh] OR “chronic kidney disease”[All Fields] OR “chronic renal disease”[All Fields] OR “chronic renal insufficiency”[All Fields] OR “CKD”[All Fields] OR “end-stage renal disease”[All Fields] OR “chronic kidney failure”[All Fields] OR “chronic renal failure”[All Fields] OR “chronic kidney diseases”[All Fields] OR “chronic renal diseases”[All Fields] OR “chronic renal insufficiencies”[All Fields] OR “end-stage renal diseases”[All Fields] OR “chronic kidney failures”[All Fields] OR “chronic renal failures”[All Fields] OR “stroke”[Mesh] OR “stroke”[All Fields] OR “strokes”[All Fields] OR “brain vascular accident”[All Fields] OR “brain vascular accidents”[All Fields] OR “apoplexy”[All Fields] OR "cerebrovascular accident”[All Fields] OR "cerebrovascular accidents”[All Fields] OR “cardiomyopathies”[Mesh] OR “cardiomyopathy”[All Fields] OR “cardiomyopathies”[All Fields] OR "myocardial disease”[All Fields] OR "myocardial diseases”[All Fields] OR “myocardiopathy”[All Fields] OR “myocardiopathies”[All Fields] OR “heart neoplasms”[Mesh] OR “heart neoplasm”[All Fields] OR “heart neoplasms”[All Fields] OR "cardiac tumor”[All Fields] OR "cardiac tumors”[All Fields] OR "myocardial tumor”[All Fields] OR "myocardial tumors”[All Fields] OR "cardiac carcinoma”[All Fields] OR "cardiac carcinomas”[All Fields] OR "heart cancer”[All Fields] OR "cardiac cancers”[All Fields] OR "cardiac cancer”[All Fields] OR "heart tumor”[All Fields] OR "heart tumors”[All Fields] OR “myocardial ischemia”[Mesh] OR “myocardial ischemia”[All Fields] OR “myocardial ischemias”[All Fields] OR "ischemic heart disease”[All Fields] OR "ischemic heart diseases”[All Fields] OR “myocardial ischaemia”[All Fields] OR “myocardial ischaemias”[All Fields] OR "ischaemic heart disease”[All Fields] OR "ischaemic heart diseases”[All Fields] OR “acute coronary syndrome”[All Fields] OR “acute coronary syndromes”[All Fields] OR “coronary disease”[All Fields] OR “coronary diseases”[All Fields] OR “coronary artery disease”[All Fields] OR “coronary artery diseases”[All Fields] OR “coronary arteriosclerosis”[All Fields] OR “Coronary atherosclerosis”[All Fields] OR “coronary stenosis”[All Fields] OR “coronary stenoses”[All Fields] OR “coronary restenosis”[All Fields] OR “coronary restenoses”[All Fields] OR “coronary heart disease”[All Fields] OR “coronary heart diseases”[All Fields] OR “coronary thrombosis”[All Fields] OR “coronary thromboses” OR “coronary occlusion”[All Fields] OR “coronary occlusions”[All Fields] OR “myocardial infarction”[All Fields] OR “myocardial infarctions”[All Fields] OR “heart attack”[All Fields] OR “heart attacks”[All Fields] OR “myocardial infarct”[All Fields] OR “myocardial infarcts”[All Fields] OR “heart arrest”[Mesh] OR “heart arrest”[All Fields]

OR “heart arrests”[All Fields] OR “cardiac arrest”[All Fields] OR “cardiac arrests”[All Fields] OR “asystole”[All Fields] OR “asystoles”[All Fields] OR “cardiopulmonary arrest”[All Fields] OR “cardiopulmonary arrests”[All Fields] OR

“heart failure”[Mesh] OR “heart failure”[All Fields] OR “heart failures”[All Fields] OR “cardiac failure”[All Fields] OR “cardiac failures”[All Fields] OR “myocardial failure”[All Fields] OR “myocardial failures”[All Fields] OR “heart decompensation”[All Fields] OR “hypertension”[Mesh] OR “hypertension”[All Fields] OR “hypertensions”[All Fields] OR “high blood pressure”[All Fields] OR “high blood pressures”[All Fields] OR “cardiovascular diseases”[Mesh] OR “cardiovascular disease”[All Fields] OR “cardiovascular diseases”[All Fields] OR "cardiovascular risk”[All Fields] OR "cardiovascular risks”[All Fields] OR “salt” [All Fields] OR “tobacco” [All Fields] OR “physical activity” [All Fields] OR “diet”[All Fields]

Task-Shifting: ((“Task”[All Fields] OR "tasks"[all fields]) AND ("shift"[All fields] OR "share"[All fields] OR "shifted"[all fields] OR "shifts"[all fields] OR "shifting"[all fields])) OR (shortage*[All Fields] AND ("physicians"[MeSH] OR “health personnel”[Mesh] OR "physicians"[All Fields] OR "doctors"[All Fields] OR "trained personnel"[All Fields] OR "health workforce"[All Fields] OR “health care workforce"[All Fields] OR “healthcare workforce"[All Fields] OR "health workers"[All Fields] OR "health care workers"[All Fields] OR “healthcare workers"[All Fields] OR "health care providers"[All Fields] OR “health providers"[All Fields] OR “healthcare providers"[All Fields])) OR ("nurse led"[All Fields] OR "primary health care nurse"[All Fields] OR "primary health care nurses"[All Fields] OR "primary health care nursing"[All Fields]) OR "nonphysician clinicians"[All Fields] OR "non-physician clinicians"[All Fields] OR “non physician health care workers"[All Fields] OR “nonphysician health care workers"[All Fields] OR “non physician health care worker"[All Fields] OR “nonphysician healthcare workers"[All Fields] OR “nonphysician health workers"[All Fields] OR “non physician health workers"[All Fields] OR ("role"[All Fields] AND ("nurse"[All Fields] OR "nurses"[all fields] OR "nursing"[all fields])) OR “community health aides”[All Fields] OR “community health centers”[mesh] OR “lay health workers"[All Fields] OR “lay health care workers"[All Fields] OR “lay health care worker"[All Fields] OR “community health workers"[All Fields] OR “community health care workers"[All Fields] OR “community healthcare workers"[All Fields] OR “community health center”[All Fields] OR “community Health centers”[all fields] OR “community health centre”[All fields] OR “community health centres”[All Fields] OR “extended scope practitioner”[all fields] OR “extended scope practitioners”[all fields] OR “extended scope practice”[all fields] OR “enhanced role”[all fields] OR “role enhancement”[all fields] OR ((“substitution”[All Fields] OR “substituted”[All Fields] OR “substitute”[All Fields] OR “substituting”[All Fields] OR “substitutes”[All Fields] OR “delegate”[All Fields] OR “delegating”[All Fields] OR “delegates”[All Fields] OR “delegation”[All Fields] OR “delegated”[All Fields]) AND (“physicians”[mesh] OR “physician”[All Fields] OR “physicians”[All Fields] OR “doctor”[All Fields] OR “doctors”[All Fields]))

Low-and Middle-income countries: "developing countries"[all fields] OR "developing country"[all fields] OR "developing countries"[mesh] OR “medically underserved area”[mesh] OR “medically underserved area”[all fields] OR “medically underserved areas”[all fields] OR “low income countries”[all fields] OR “low income country”[all fields] OR “middle income countries”[all fields] OR “middle income country”[all fields] OR “global”[all fields] OR “resource poor”[all fields] OR “low resource”[all fields] OR “Africa”[Mesh] OR “Asia, Central”[Mesh] OR “Asia, Western”[Mesh] OR “Asia, Southeastern”[Mesh] OR “Indian Ocean Islands”[Mesh] OR “Central America”[Mesh] OR “South America”[Mesh] OR “Europe, Eastern”[Mesh] OR “Transcaucasia”[Mesh] OR “China”[Mesh] OR “Korea”[Mesh] OR “Mongolia”[Mesh] OR “Mexico”[Mesh] OR “Caribbean Region”[Mesh] OR “Pacific Islands”[Mesh] OR “Africa”[all fields] OR “Central Asia”[all fields] OR “western Asia”[all fields] OR “southeastern Asia”[all fields] OR “Indian Ocean Islands”[all fields] OR “Central America”[all fields] OR “South America”[all fields] OR “eastern Europe”[all fields] OR “Transcaucasia”[all fields] OR “Caribbean”[all fields] OR “Pacific Islands”[all fields] OR “Afghan”[all fields] OR “afghani”[all fields] OR “afghanistan”[all fields] OR “Bangladesh”[all fields] OR “bangladeshi”[all fields] OR “Benin”[all fields] OR “Beninese”[all fields] OR “Burkina Faso”[all fields] OR “Burkinabe”[all fields] OR “Burundi”[all fields] OR “burundian”[all fields] OR “Cambodia”[all fields] OR “cambodian”[all fields] OR “Central African Republic”[all fields] OR “central African”[all fields] OR “Chad”[all fields] OR “chadian”[all fields] OR “Comoros”[all fields] OR “comoran”[all fields] OR “Congo”[all fields] OR “congolese”[all fields] OR “Eritrea”[all fields] OR “eritrean”[all fields] OR “Ethiopia”[all fields] OR “ethiopian”[all fields] OR “Gambia”[all fields] OR “gambian”[all fields] OR “Guinea”[all fields] OR “guinean”[all fields] OR “Haiti”[all fields] OR “haitian”[all fields] OR “Kenya”[all fields] OR “Kenyan” OR “Korea”[all fields] OR “korean”[all fields] OR “Kyrgyz”[all fields] OR "kyrgyzstan"[all fields] OR “Liberia”[all fields] OR “liberian”[all fields] OR “Madagascar”[all fields] OR “malagasy”[all fields] OR “Malawi”[all fields] OR “malawian”[all fields] OR “mali”[all fields] OR “malian”[all fields] OR “mozambique”[all fields] OR “mozambican”[all fields] OR “Myanmar”[all fields] OR “myanmarese”[all fields] OR “burmese”[all fields] OR “Nepal”[all fields] OR “Nepalese”[all fields] OR “Niger”[all fields] OR “nigerian”[all fields] OR “Rwanda”[all fields] OR “rwandan”[all fields] OR “Sierra Leone”[all fields] OR “sierra leonean”[all fields] OR “Somalia”[all fields] OR “somalian”[all fields] OR “Tajikistan”[all fields] OR “tajik”[all fields] OR “tadzhik”[all fields] OR” Tanzania”[all fields] OR “tanzanian”[all fields] OR

“Togo”[all fields] OR “togolese”[all fields] OR “Uganda”[all fields] OR “ugandan”[all fields] OR “Zimbabwe”[all fields] OR “zimbabwean”[all fields] OR “Angola”[all fields] OR “angolan”[all fields] OR “Armenia”[all fields] OR “armenian”[all fields] OR “Belize”[all fields] OR “belizean”[all fields] OR “Bhutan”[all fields] OR “bhutanese”[all fields] OR “Bolivia”[all fields] OR “bolivian”[all fields] OR “Cameroon”[all fields] OR “cameroonian”[all fields] OR “Cape Verde”[all fields] OR “cape verdian”[all fields] OR “cape verdean”[all fields] OR “Côte d'Ivoire” [all fields] OR "ivory coast"[all fields] “ivorian”[all fields] OR “Djibouti”[all fields] OR “Egypt”[all fields] OR “egyptian”[all fields] OR “El Salvador”[all fields] OR “salvadoran”[all fields] OR “Fiji”[all fields] OR “fijian”[all fields] OR “Georgia”[all fields] OR “georgian”[all fields] OR “Ghana”[all fields] OR “ghanaian”[all fields] OR “Guatemala”[all fields] OR “Guatemalan”[all fields] OR “Guyana”[all fields] OR “guyanese”[all fields] OR “Honduras” OR “honduran”[all fields] OR “Indonesia”[all fields] OR “indonesian”[all fields] OR “India”[all fields] OR “indian”[all fields] OR “Iraq”[all fields] OR “iraqi”[all fields] OR “Kiribati”[all fields] OR “Kosovo”[all fields] OR “kosovar”[all fields] OR “Laos”[all fields] OR “lao”[all fields] OR “laotian”[all fields] OR “Lesotho”[all

fields] OR “Marshall Islands”[all fields] OR “marshallese”[all fields] OR “Mauritania”[all fields] OR “mauritanian”[all fields] OR “Micronesia”[all fields] OR “micronesian”[all fields] OR “Moldova”[all fields] OR “moldovan”[all fields] OR “Mongolia”[all fields] OR “mongolian”[all fields] OR “Morocco”[all fields] OR “moroccan”[all fields] OR “Nicaragua”[all fields] OR “nicaraguan”[all fields] OR “Nigeria”[all fields] OR “nigerian”[all fields] OR “Pakistan”[all fields] OR “pakistani”[all fields] OR “Papua New Guinea”[all fields] OR “papua new guinean”[all fields] OR “Paraguay”[all fields] OR “paraguayan”[all fields] OR “Philippines”[all fields] OR “filipino”[all fields] OR “Samoa”[all fields] OR “samoan”[all fields] OR “Sao Tome and Principe”[all fields] OR "São Tomé and Príncipe"[all fields] OR “Senegal”[all fields] OR “senegalese”[all fields] OR “Solomon Islands”[all fields] OR “Solomon islander”[all fields] OR “Sri Lanka”[all fields] OR “sri lankan”[all fields] OR “Sudan”[all fields] OR “sudanese”[all fields] OR “Swazi”[all fields] OR “swaziland”[all fields] OR “Syria”[all fields] OR “syrian”[all fields] OR “east Timor”[all fields] OR “east timorese”[all fields] OR “Tonga”[all fields] OR “tongan”[all fields] OR “Turkmenistan”[all fields] OR “turkmen”[all fields] OR “Tuvalu”[all fields] OR “tuvaluan”[all fields] OR “Ukraine”[all fields] OR “ukrainian”[all fields] OR “Uzbekistan”[all fields] OR “uzbek”[all fields] OR “Vanuatu”[all fields] OR “Vietnam”[all fields] OR “vietnamese”[all fields] OR “West Bank”[all fields] OR “Gaza”[all fields] OR “Yemen”[all fields] OR “yemeni”[all fields] OR “yemenite”[all fields] OR “Zambia”[all fields] OR “zambian”[all fields] OR “Albania”[all fields] OR “albanian”[all fields] OR “Algeria”[all fields] OR “algerian”[all fields] OR “Antigua and Barbuda”[all fields] OR “antiguan”[all fields] OR “Barbuda”[all fields] OR “Argentina”[all fields] OR “argentinian”[all fields] OR “Azerbaijan”[all fields] OR “azerbaijani”[all fields] OR “Belarus”[all fields] OR “belarusian”[all fields] OR “Bosnia”[all fields] OR “bosnian”[all fields] OR “Botswana”[all fields] OR “Brazil”[all fields] OR “brazilian”[all fields] OR “Bulgaria”[all fields] OR “bulgarian”[all fields] OR “Chile”[all fields] OR “chilean”[all fields] OR “China”[all fields] OR “Chinese”[all fields] OR “Colombia”[all fields] OR “colombian”[all fields] OR “Costa Rica”[all fields] OR “costa rican”[all fields] OR “Cuba”[all fields] OR “Cuban”[all fields] OR “Dominica”[all fields] OR “dominican”[all fields] OR “Ecuador”[all fields] OR “ecuadorean”[all fields] OR “Gabon”[all fields] OR “gabonese”[all fields] OR “Grenada”[all fields] OR “grenadian”[all fields] OR “Iran”[all fields] OR “iranian”[all fields] OR “Jamaica”[all fields] OR “jamaican”[all fields] OR “Jordan”[all fields] OR “jordanian”[all fields] OR “Kazakhstan”[all fields] OR “kazakhstani”[all fields] OR “Latvia”[all fields] OR “latvian”[all fields] OR “Lebanon”[all fields] OR “lebanese”[all fields] OR “Libya”[all fields] OR “libyan”[all fields] OR “Lithuania”[all fields] OR “lithuanian”[all fields] OR “Macedonia”[all fields] OR “macedonian”[all fields] OR “Malaysia”[all fields] OR “malaysian”[all fields] OR “Maldives”[all fields] OR “maldivian”[all fields] OR “mauritius”[all fields] OR “mauritian”[all fields] OR “Mexico”[all fields] OR “mexican”[all fields] OR “Montenegro”[all fields] OR “montenegrin”[all fields] OR “Namibia”[all fields] OR “namibian”[all fields] OR “Palau”[all fields] OR “palauan”[all fields] OR “Panama”[all fields] OR “panamanian”[all fields] OR “Peru”[all fields] OR “peruvian”[all fields] OR “Romania”[all fields] OR “romanian”[all fields] OR “Russia”[all fields] OR “russian”[all fields] OR “Serbia”[all fields] OR “serbian”[all fields] OR “Seychelles”[all fields] OR “seychellois”[all fields] OR “South Africa”[all fields] OR “south african”[all fields] OR “Saint Kitts”[all fields] OR “saint Lucia”[all fields] OR “Saint Vincent”[all fields] OR “Suriname”[all fields] OR “surinamer”[all fields] OR “thailand”[all fields] OR “Thai”[all fields] OR “Tunisia”[all fields] OR “tunisian”[all fields] OR “Turkey”[all fields] OR “turkish”[all fields] OR “Uruguay”[all fields] OR “uruguayan”[all fields] OR “Venezuala”[all fields] OR “venezualan”[all fields] OR “Sub Saharan Africa”[all fields]

Database: EMBASE(OVID)

Run on 18 August 2017

	Searches	Results
1	(hyperlipidemia or hyperlipidaemia$ or hyperlipemia$1 or lipidaemia$1 or high cholesterol or hypercholesteroleamia$1 or hypercholesteraemia$1 or Diabetes mellitus or diabetes or diabetic angiopathy or diabetic or hyperglycemia$1 or proteinuria or proteinuria$1 or albuminuria$1 or hemoglobinuria$1 or chronic kidney disease or chronic kidney disease$1 or chronic renal disease$1 or chronic renal insufficienc$ or CKD or endstage renal disease$1 or chronic kidney failure$1 or chronic renal failure$1 or stroke or stroke$1 or brain vascular accident$1 or apoplexy or cerebrovascular accident$1 or myocardial disease or cardiomyopath$ or myocardial disease$1 or myocardiopath$ or heart muscle ischaemia$1 or myocardial ischemia$1 or ischaemic heart disease$1 or acute coronary syndrome$1 or coronary disease$1 or coronary artery disease$1 or coronary arterioscleros$ or coronary atheroscleros$ or coronary stenos$ or coronary restenos$ or coronary heart disease$1 or coronary thrombos$ or coronary occlusion$1 or myocardial infarct$ or heart attack$1 or heart tumor or heart neoplasm$1 or cardiac tumor$1 or myocardial tumor$1 or cardiac carcinoma$1 or heart cancer$1 or cardiac cancer$1 or heart tumor$1 or heart failure or heart arrest$1 or cardiac arrest$1 or asystole$1 or cardiopulmonary arrest$1 or heart failure$1 or cardiac failure$1 or myocardial failure$1 or heart decompensation$1 or hypertension or hypertension$1 or high blood pressure$1 or cardiovascular disease or cardiovascular risk or (cardiovascular adj 5 disease$1) or (cardiovascular adj 5 risk$1)).af.	2987525
2	(personnel shortage or (shortage$ adj5 doctor$) or (shortage$ adj5 physician$) or (shortage$ adj5 trained adj5 personnel) or (shortage$ adj5 health adj5 workforce) or (shortage$ adj5 health adj5 worker$) or (shortage$ adj5 health adj5 provider$) or (task$ adj5 shift$) or non$ physician clinician$ or non$ physician health$ worker$ or primary health care nurs$ or (role adj5 nurs$) or	74784

	community health nursing or health auxiliary or community health$ worker$ or community health cent$ or lay health$ worker$ or community health$ aide$ or (community adj2 health adj5 worker$) or extended scope practi$ or (role adj3 enhance$) or (substitut$ adj10 physician$) or (substitut$ adj10 doctor$) or (substitute$ adj10 nurse$) or (delegat$ adj10 physician$) or (delegat$ adj10 doctor$) or (delegat$ adj10 nurse$)).af.	
3	(Uganda$ or Zimbabwe$ or Angola$ or Armenia$ or Beliz$ or Bhutan$ or Bolivia$ or Cameroon$ or Cape Verde$ or Congo$ or "Côte d'Ivoire" or Ivory Coast or Ivorian or Djibouti or Egypt$ or El Salvador or Salvadoran or Fiji$ or Georgia$ or Ghana$ or Guatemala$ or Guyan$ or Hondura$ or Indonesia$ or India$ or Iraq$ or Kiribati or Kosov$ or Lao$ or Lesotho or Marshall Islands or Marshallese or Mauritania$ or Micronesia$ or Moldov$ or Mongolia$ or Morocc$ or Nicaragua$ or Nigeria$ or Pakistan$ or Papua New Guinea$ or Paraguay$ or Philippines or Filipino or Samoa$ or sao tome$ or Senegal$ or Solomon Island$ or sri lanka$ or Sudan$ or Swazi$ or Syria$1or Timor$ or Tonga$1or Turkmen$ or Tuvalu$ or Ukrain$ or Uzbek$ or Vanuat$ or Vietnam$ or West Bank or Gaza or Yemen$ or Zambia$ or Albania$ or Algeria$ or "Antigua and Barbuda" or antiguan or barbudan or Argentin$ or Azerbaijan$ or Belarus$ or Bosnia$ or Botswana or Brazil$ or Bulgaria$ or Chile$ or Chinaor Chinese or Colombia$ or Costa Rica$ or Cuba$ or Dominica$ or Ecuador$ or Gabon$ or Grenad$ or Iran$ or Jamaica$ or Jordan$ or Kazakhstan$ or Latvia$ or Leban$ or Libya$ or Lithuania$ or Macedonia$ or Malaysia$ or Maldiv$ or mauriti$ or Mexic$ or Montenegr$ or Namibia$ or Palau$ or Panama$ or Peru$ or Romania$ or Russia$ or Serbia$ or Seychell$ or South Africa$ or Saint Kitts or Saint Lucia or Saint Vincent or Suriname$ or Thai$ or Tunisia$ or Turk$ or Uruguay$ or Venezuala$ or "Sub Saharan Africa$1").af.	4767627
4	1 and 2 and 3	909
5	limit 4 to em=197401-201732	906

Database: CINAHL(EBSCO)

Run on 31st August 2017

	Search Terms	Search Options	Search results
S5	#S4	Limiters - Published Date: 19370101-20170831	(288)
S4	S1 AND S2 AND S3		(1,715)
S3	TX (MH "developing countries+") OR (MH "medically underserved area+") OR (TX "developing countr*") OR (TX "medically underserved area#") OR (TX "low income countr *") OR (TX "middle income countr*") OR (MH "Africa+") OR (TX Africa#) OR (TX Caribbean) OR (MH "west indies+") OR (TX "central America#") OR (MH "Central America+") OR (TX "south America#") OR (MH "south America+") OR (TX global) OR (TX "low resource") OR (TX "resource poor") OR (TX "central asia#") OR (MH "asia, central+") OR (TX "sou ...		(585,321)
S2	TX (MH "Personnel Shortage+") OR (TX shortage# N5 doctor#) OR (TX shortage# N5 physician#) OR (TX shortage# N5 "trained personnel") OR (TXshortage# N5 "health* * workforce") OR (TX shortage# N5 "health* * worker#") OR (TX shortage# N5 "health* * provider#") OR (TX task# N5 shift*) OR (TX "nurse led") OR (TX "non*physician clinicians") OR (TX "non*physician health* * worker#") OR (TX "primary health* * nurs*") OR (TX role N5 nurs*) OR (MH "community health workers+") OR (MH "community health cent ...		(89,482)

S1	TX (MH “cardiovascular diseases+”) OR (TX cardiovascular N5 disease#) OR (MH “cardiovascular risk factors+”) OR (TX cardiovascular N5 risk#) OR (MH “Hyperlipidemia+”) OR (TX hyperlipid#emia#) OR (TX hyperlip#emia#) OR (TX lipid#emia#) OR (TX "high cholesterol") OR (TX hypercholesterol#emia#) OR (TX hypercholester#emia#) OR (TX diabetes) OR (TX diabetic)OR(TX hyperglyc#emia#) OR (MH“Diabetes Mellitus+”) OR (MH “proteinuria+”) OR (TX proteinuria#) OR (TX albuminuria#) OR (TX hemoglobinuria#) OR (M ...		(453,686)

Box 2: Statistical methods used in the meta-analysis

We resolved the “unit of analyses” issues in cluster RCT by estimating effective sample size for each study. To calculate the effective sample size, we used the following method.

Effective sample size for intervention arm= Original sample size in intervention arm of the study/design effect

Effective sample size for control arm= Original sample size in control arm of the study/design effect

We calculated design effect using the formula “Design effect=1+(M-1)*ICC” where “M”- Average cluster size; “ICC”- intra-cluster correlation coefficient. All the cluster RCT in our meta-analysis has provided ICC either in result publication or in cRCT protocol publication except Sarfo et al from Ghana. We assumed ICC from another study conducted Ghana.[1] (Ogedegbe G et al. A cluster-randomized trial of task shifting and blood pressure control in Ghana: study protocol. *Implementation Scince*.2014;9(1):73.

Ogedegbe et al 2018 :

Design effect= 1+(M-1)ICC

M→ Average cluster size (total individual/total cluster number)

ICC→ intra cluster correlation coefficient

M= (757/32)=23.66 ; ICC=0.06

Design effect= 1+(23.66-1)*0.06=2.35

Effective sample size in HIC (Control) =389/2.35=165.53~166

Effective sample size TASSH (Intervention) = 368/2.35=156.59~157
